# σ-Bond Electron Delocalization in Oligosilanes as Function of Substitution Pattern, Chain Length, and Spatial Orientation

**DOI:** 10.3390/molecules21081079

**Published:** 2016-08-18

**Authors:** Johann Hlina, Filippo Stella, Mohammad Aghazadeh Meshgi, Christoph Marschner, Judith Baumgartner

**Affiliations:** 1 Institut für Anorganische Chemie, Technische Universität Graz, Stremayrgasse 9, A-8010 Graz, Austria; johann.hlina@tugraz.at (J.H.); filippo.stella@unipd.it (F.S.); m.meshgi@ucc.ie (M.A.M.); 2 Institut für Chemie, Karl Franzens Universität Graz, Stremayrgasse 9, 8010 Graz, Austria

**Keywords:** oligosilanes, σ-bond electron delocalization, UV-spectroscopy, single crystal diffraction analysis

## Abstract

Polysilanes are known to exhibit the interesting property of σ-bond electron delocalization. By employing optical spectroscopy (UV-vis), it is possible to judge the degree of delocalization and also differentiate parts of the molecules which are conjugated or not. The current study compares oligosilanes of similar chain length but different substitution pattern. The size of the substituents determines the spatial orientation of the main chain and also controls the conformational flexibility. The chemical nature of the substituents affects the orbital energies of the molecules and thus the positions of the absorption bands.

## 1. Introduction

Polysilanes, that is compounds containing Si-Si bonds, are known to exhibit optical properties similar to conjugated organic molecules [1]. However, in these compounds, it is not π-electrons that are distributed over a system of multiple bonds, but σ-electrons that are delocalized over a number of σ-bonds. For both types of delocalization it is important that the involved orbitals are spatially aligned in a way that they can overlap. For conjugated π-systems, this means that all involved atoms are located in one plane and for delocalized σ-electrons this requirement is associated with *anti-* or *transoid* conformations of the involved molecular chain [2,3].

The σ electron delocalization effect was initially discovered by the research groups of Kumada [4,5,6] and Gilman [7,8] in the 1960s, in the course of studying UV/vis spectra of small oligosilanes. Later, longer polysilanes were found to exhibit thermochromism, where the length of aligned chain segments which absorb light of a certain wave length, changes as a function of temperature [9].

Our own studies in this field [10,11,12,13,14,15,16,17] are strongly connected to investigations concerned with the chemistry of silanides [18]. The availability of polysilanyl anions allows the construction of oligosilanes chains with defined conformational properties.

We were thus interested in studying whether it is possible to impose preferred conformations on short oligosilanes by the deliberate choice of substitution pattern. By careful variation of substituent type and shape, it should be possible to manipulate the σ-electron delocalization.

## 2. Results

**Figure 1:**
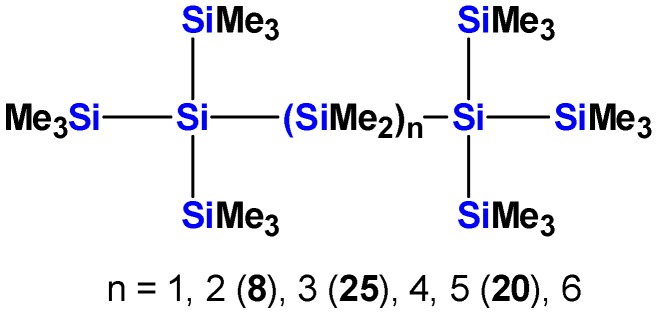
Oligosilanes with bulky tris(trimethylsilyl)silyl end groups.

In our previous studies we learned that, while a rotational process in permethylated oligosilanes is energetically very facile, the introduction of bulky end groups such as the tris(trimethylsilyl)silyl unit (Figure 1) substantially suppresses these rotations if the groups are not too far apart [10]. This is consistent with the appearance of only one low energy UV-absorption band, which can be associated with an *all*-*transoid* conformer. With increasing chain length between two tris(trimethylsilyl)silyl units, their steric interaction diminishes and thus the force that drives the molecule to engage only in one conformer diminishes. This effect is reflected in the UV-absorption spectra by the appearance of a second low energy absorption band of higher energy for internal α,ω-silanylene chain units of -(SiMe_2_)_5_- and longer, which corresponds to a conformer with a shorter *transoid* aligned segment [10].

**Figure 2:**
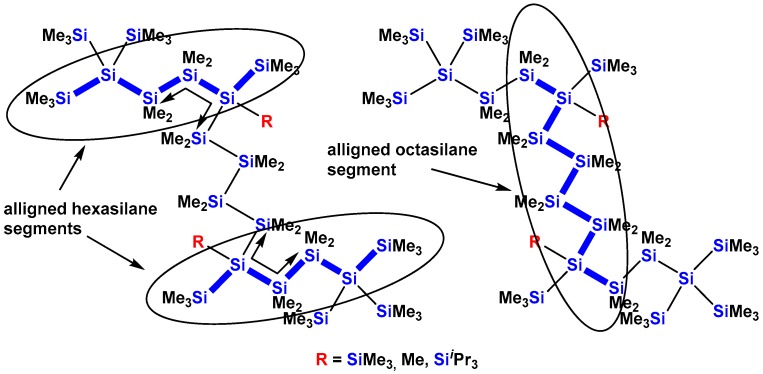
Schematic representation of the preferentially existing conformer of a permethylated tetradecasilane with geminal trimethylsilyl substitution at the 2, 5, 10, and 13 positions. Terminal *transoid* aligned hexasilane segments marked on the left side, a *transoid* aligned octasilane segment on the right side.

In a subsequent study, we found that for longer chains with (Me_3_Si)_3_Si end groups, the formal substitution of two geminal methyl groups on internal -(SiMe_2_)- units for trimethylsilyl groups enforces the ability of the molecule to exist as a single conformer again by suppressing rotational processes. However, the thus favored conformers were found to not be *all-transoid* ones but consist of several *transoid* aligned segments which are separated by the introduced trimethylsilyl groups [13]. This behavior is exemplified in Figure 2. Additional information about conformational preferences of oligosilanes was obtained by single crystal X-ray diffraction analysis. Of course, the solid state structure of a polysilane is not necessarily identical with the structure in solution. For the cases of longer chains with a preference for only one conformer, Raman spectroscopic measurements of solution and solid state samples suggest identical conformational properties [10].

The current study can be considered as a continuation of previous work [10,13,14,15,16,17]. In a first part, the comparison of the UV absorption spectra of a set of hexasilanes with different substitution patterns allows a judgement of substituent influence on orbital energies and conformational properties. A second part deals with longer oligosilanes which are variations of the previously investigated compounds where trimethylsilyl groups are either exchanged for methyl or for triisopropylsilyl groups. A final small part is dedicated to UV-spectroscopic analysis of some cyclosilanes.

### 2.1. Synthesis of Oligosilanes

**Scheme 1:**
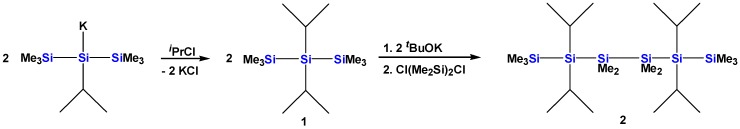
Synthesis of a hexasilane with bulky isopropyl groups in the 2- and 5-positions.

Compound **1** was obtained by simple reaction of isopropylbis(trimethylsilyl)silylpotassium [19] with isopropylchloride (Scheme 1). It is interesting to note that **1** reacts cleanly with potassium *tert-*butoxide to the respective 1,1-diisopropyltrimethyldisilanylpotassium. The attempt to achieve similar chemistry with octamethyltrisilane led to complicated oligomerization chemistry [18,20]. A likely reason for the clean conversion of **1** seems to be the increased steric demand of 1,1-diisopropyltrimethylsilanylpotassium compared to pentamethyldisilanylpotassium. Further reaction with 1,2-dichlorotetramethydisilane gave compound **2** (Scheme 1).

**Scheme 2:**
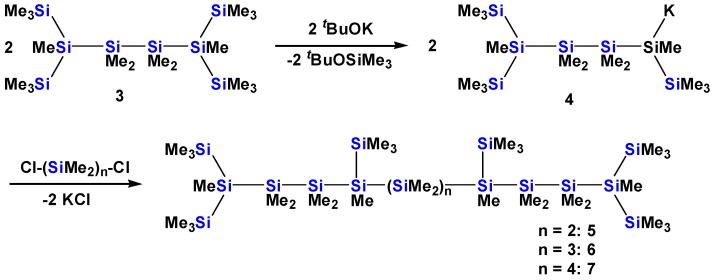
Synthesis of elongated oligosilanes with trimethylsilyl substituents in the core and at the periphery of the chains.

**Figure 3:**
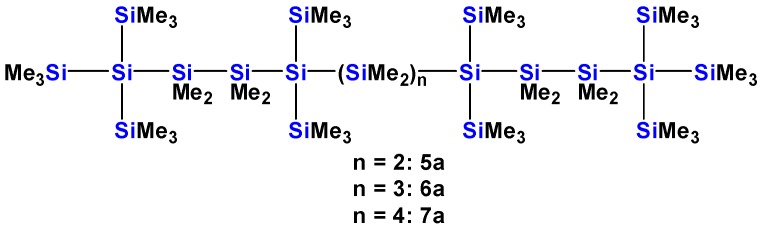
Compounds **5a**, **6a**, and **7a** serve as reference standards for the judgement of the optical properties of the two series of oligosilanes **5**, **6**, and **7** as well as **11**, **12**, and **13**.

Reaction of 2,5-bis(trimethylsilyl)decamethylhexasilane (**3**) with potassium *tert*-butoxide gives the silanide **4** in a clean reaction (Scheme 2). The latter can be used as building block for the construction of a series of oligosilane chains. Coupling of 2 equiv. of **4** with 1,2-dichlorotetramethyldisilane, 1,3-dichlorohexamethyltrisilane, or 1,4-dichlorooctamethyltetrasilane gave compounds **5**, **6**, and **7** with **12**, **13**, and **14** catenated silicon atoms in the main chain (Scheme 2). The strategy to prepare **5**, **6**, and **7** is similar to what we have reported earlier for the synthesis of **5a**, **6a,** and **7a** (Figure 3) [13].

The latter were prepared from 2,2,5,5-tetrakis(trimethylsilyl)decamethylhexasilane (**8**) and contain tris(trimethylsilyl)silyl end groups and bis(trimethylsilyl)silylene units in the main chain, which were found to cause a turn in the main chain, interrupting an *all-transoid* conformation.

**Scheme 3:**
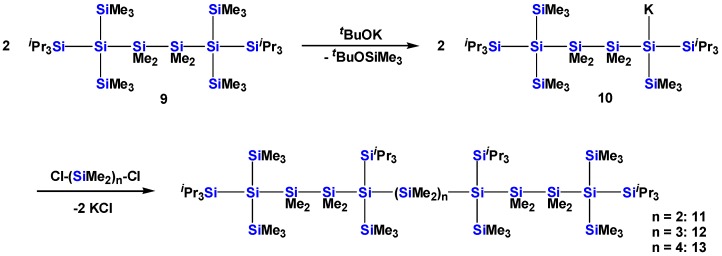
Synthesis of elongated oligosilanes with trimethylsilyl and triisopropylsilyl substituents in the core and at the periphery of the chains.

Another strategy for the manipulation of conformational properties of oligosilanes chains aims in the opposite direction. Instead of replacing trimethylsilyl units by methyl groups and thus creating more flexible chains, it is possible to increase the steric influence of substituents by replacing trimethylsilyl groups with triisopropylsilyl groups. Starting from 2,5-bis(triisopropylsilyl)-2,5-bis(trimethylsilyl)decamethylhexasilane (**9**) reaction with potassium *tert*-butoxide gives the respective silanide **10** in a clean reaction (Scheme 3). Two equiv. of **10** can be coupled to the elongated oligosilanes **11**, **12**, and **13** (Scheme 3).

**Scheme 4:**
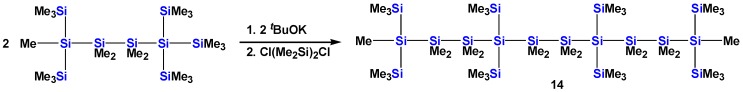
Synthesis of a dodecasilane chain with two internal bis(trimethylsilyl)silylene units and bis(trimethylsilyl)methylsilyl end groups.

Compound **14** represents an oligosilanes chain which contains bis(trimethylsilyl)silylene units in the core of the chain as in the compounds of the previous study but features bis(trimethylsilyl)methylsilyl end groups instead of tris(trimethylsilyl)silyl groups. The compound was prepared by reaction of 2,5-bis(trimethylsilyl)undecamethylhexasilanyl-2-potassium with 1,2-dichlorotetramethyldisilane (Scheme 4).

**Scheme 5:**
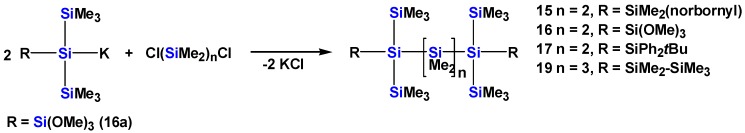
Synthesis of a number of oligosilanes with derivatized tris(trimethylsilyl)silyl groups and α,ω-oligosilanylene units of different lengths.

**Figure 4:**
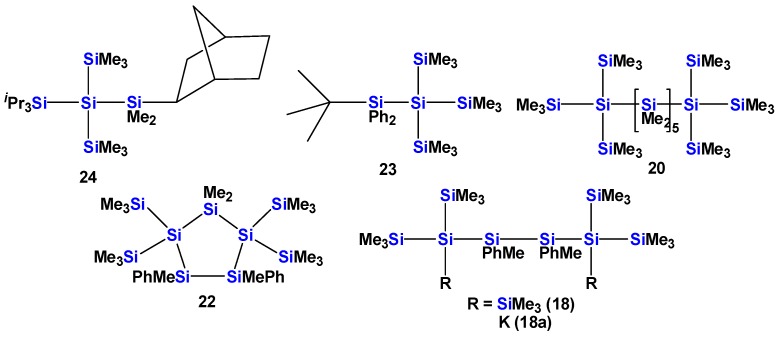
Some new compounds prepared in this study either as starting materials or as subjects for UV-spectroscopic studies.

In our studies, we found that the simply accessible 2,2,5,5-tetrakis(trimethylsilyl)decamethylhexasilane (**8**) is a conformationally rather rigid molecule. Since analogous compounds are easily available, it was tempting to prepare a number of compounds of molecules with different end groups (**15**, **16**, **17**) (Scheme 5). Compound **18** with phenyl groups on the 1,2-disilanylene unit (Figure 4) was synthesized in a similar way by reaction of tris(trimethylsilyl)silyl potassium with the 1,2-ditriflate obtained from 1,2-dimethyltetraphenyldisilane. Also, compound **19** was prepared in a similar way (Scheme 5) and was then converted further to compound **20** by AlCl_3_ catalyzed isomerization (Figure 4) [21].

**Scheme 6:**
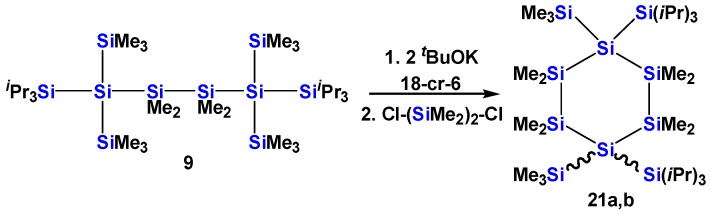
Use of 2,5-bis(trimethylsilyl)-2,5-bis(triisopropylsilyl)decamethylhexasilane for the synthesis of a mixture of *cis*- and *trans*-1,4-bis(triisopropylsilyl)-1,4-bis(trimethylsilyl)cyclohexasilane.

Compound **9** was also converted into a 1,4-disilandiide by reaction with 2 equiv. potassium *tert*-butoxide. Its reaction with 1,2-dichlorotetramethyldisilane leads to a mixture of the two isomers **21a** and **21b**, where the triisopropylsilyl groups are either *cis*- or *trans*-oriented to each other (Scheme 6). In a related way, compound **18** was converted to the 1,4-disilanide, which was further reacted with dimethyldichlorosilane to give the cyclopentasilane **22** (Figure 4).

Compound **23** (Figure 4) was prepared as starting material for the synthesis of **17** and compound **24** was used for structural comparison purposes.

### 2.2. UV-Spectroscopy Studies

UV/vis spectroscopy is a good tool to achieve some insight into the σ-electron delocalization in polysilanes. The position of the lowest energy absorption band allows making an estimate about the extension of delocalization. Effective σ-electron delocalization requires a *transoid* orientation of the polysilane chain [3,22]. If the *transoid* arrangement is broken by bending the chain into another direction, electron delocalization is interrupted. This was experimentally verified by Tsuji and Tamao in a series of elegant publications, where they were able to lock polysilane conformations by introducing a rigid backbone [23,24,25,26,27]. Our own studies [10,13,17] have shown that it is possible to prepare polysilanes with restricted rotational properties, which contain two or three different segments which exhibit σ-electron delocalization but are separated by a rotational twist. In the current study, we aim to investigate the steric and electronic influence of substituents on conformational and delocalization properties. 

**Figure 5:**
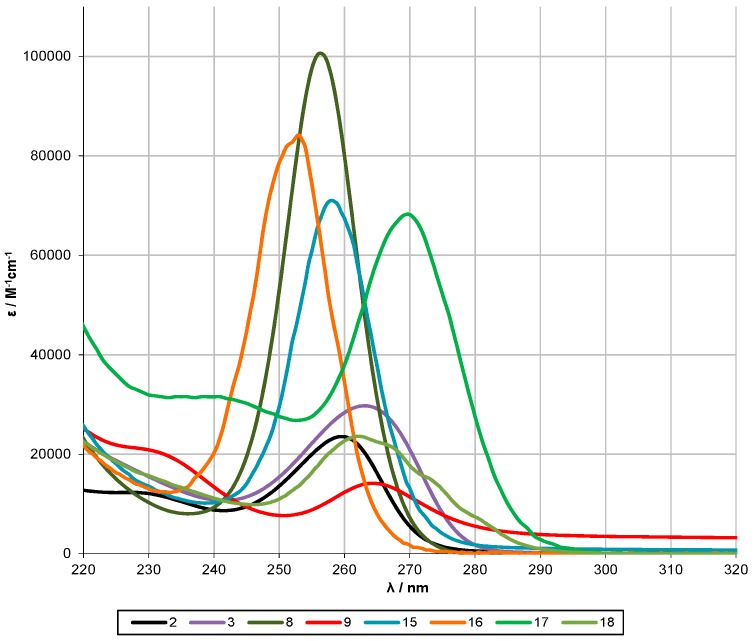
Comparison of the UV spectra of differently substituted hexasilanes.

Scheme 6 and Figure 4 show a number of hexasilanes with different substitution pattern. While it is known that the permethylated *n-*hexasilane (*n-*Si_6_Me_14_) exhibits a λ_max_ at 260 nm [6,28], the respective band for **8**, which is an *n-*hexasilane with four trimethylsilyl groups in the 2- and 5-positions, is slightly blue-shifted to 257 nm (Figure 5, Table 1) [10]. There are two possible explanations for this slight shift. On one hand, the bulk of the tris(trimethylsilyl)silyl groups largely suppresses rotation around internals Si-Si bonds and thus locks the conformation of **8** to an *all–transoid* arrangement. This enhances the absorption ability of the chain because many of the energetically accessible conformations of *n-*Si_6_Me_14_ contain segments that are not fully delocalized. On the other hand, it needs to be taken into consideration that unfavorable 1,4-interactions of the bulky tris(trimethylsilyl)silyl groups also prevent the molecule from engaging in an *all-anti* conformation. It seems, however, that this special conformation, which corresponds to a perfect σ-electron delocalization is accessible for *n-*Si_6_Me_14_. Apart from this steric explanation model, also the different electronic properties of silyl and alkyl groups should to be taken into consideration. As the σ-electron delocalization process involves σ*-orbitals of the Si-substituents bonds the energy of these is relevant.

To estimate which effect is more pronounced compounds **2**, **3**, and **9** are of interest. Compound **2**, with four bulky isopropyl groups in the 2- and 4-positions, can be considered electronically equivalent to *n-*Si_6_Me_14_ and sterically similar to **8**. The fact that the absorption maximum of **2** is identical with that of *n*-Si_6_Me_14_ seems to indicate that conformational properties of these two compounds are similar. Compound **9** is similar to **8** but two of the trimethylsilyl groups are replaced by triisopropylsilyl groups. However, the crystal structure of **9** shows a first torsional angle of w_1_ = 168° compared to 159° found for **8**. This seems to indicate a better *transoid* alignment of the chain, which results in a bathochromic shift of 7 nm to a low energy absorption maximum of 264 nm (Table 1, Figure 5). Interestingly enough, compound **3** where the same trimethylsilyl groups of **8** are replaced by methyl groups also exhibits its low energy absorption maximum at 264 nm (Table 1, Figure 5). It almost seems as if the tris(trimethylsilyl)silyl groups of **8** are particularly ill-suited for a *transoid* alignment of the chain. Even compound **15**, where two of the trimethylsilyl groups of **8** are replaced by dimethyl-*exo*-2-norbornylgroups, shows a slight bathochromic shift of the low energy absorption maximum to 258 nm (Table 1, Figure 5).

Compound **16** represents another example of formal exchange of two of the trimethylsilyl groups of **8**. This time Si(OMe)_3_ are introduced. Despite the fact that there are conformations for **16**, where the *transoid* aligned chain contains trimethylsilyl end-groups, a hypsochromic shift of the low energy absorption maximum is observed (Table 1, Figure 5). It seems evident that this is an electronic effect, caused by the less electron donating Si(OMe)_3_ groups. In contrast to this, compound **17**, where the two trimethylsilyl groups are replaced by SiPh_2_*^t^*Bu units, exhibits the most bathochromicly shifted absorption maximum at 270 nm (Table 1, Figure 5). Although the crystal structure of **17** (Table 2) does not indicate a substantially better aligned chain, it can be assumed that the phenyl groups of **17** are responsible for an extension of the delocalization. The introduction of phenyl groups into the spacer unit as can be seen in compound **18** has only a minor effect on the location of the absorption maximum (Table 1, Figure 5).

**Figure 6:**
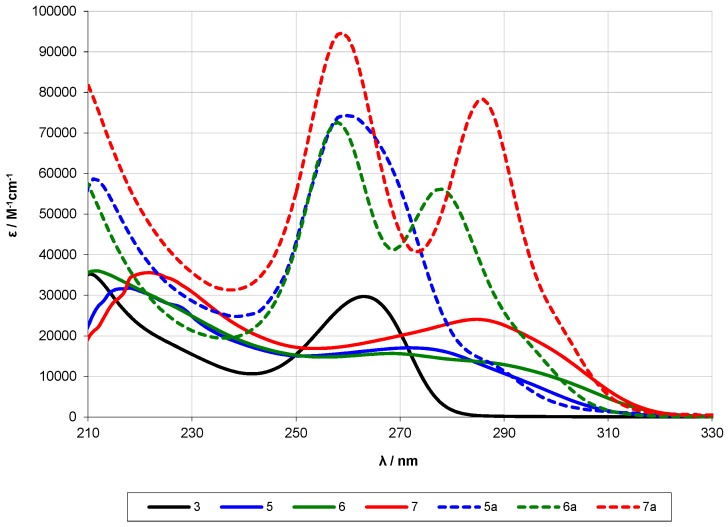
Comparison of the UV spectra of different dodeca-, trideca-, and tetradecasilanes with either methyl or trimethylsilyl groups in terminal and internal positions.

**Figure 7:**
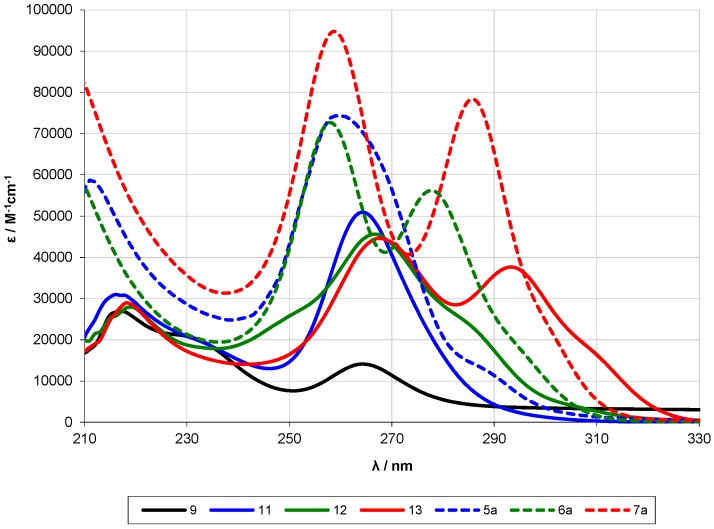
Comparison of the UV spectra of different dodeca-, trideca-, and tetradecasilanes with either triisopropylsilyl or trimethylsilyl groups in terminal and internal positions.

Figure 6 and Figure 7 provide insight to judge the effectivity of internal substituents of longer oligosilanes to control the conformation by their UV-spectra. Figure 6 shows a comparison of compounds **5**, **6**, and **7** with the respective compounds **5a**, **6a**, and **7a** [13] which possess tris(trimethylsilyl)silyl end groups and internal bis(trimethylsilyl)silylene units. The higher degree of these elements compared to bis(trimethylsilyl)methylsilyl end groups and internal trimethylsilylmethylsilylene units render **5a**, **6a**, and **7a** more rigid, whereas **5**, **6**, and **7** are more flexible and thus are able to engage in different conformations. This difference is nicely reflected in the UV spectra. The spectra of **5a** and, in particular, **6a** and **7a** display relatively sharp bands, which can be assigned to defined delocalized segments of the molecules. For **5**, **6**, and **7,** the corresponding bands are much broader indicating contributions of conformations consisting of more than two separated extended delocalized segments.

Interestingly, the picture of Figure 7 is quite different from that in Figure 6. While the latter shows the effect of increased conformational flexibility, Figure 7 features the impact of even stronger enforced conformational locking which is accomplished by exchanging some of the trimethylsilyl for triisopropylsilyl groups. A first consequence of this exchange was already visible in the comparison of **8** with **9** (Figure 5). In this case, the exchange of peripheral trimethylsilyl groups for triisopropylsilyl units resulted in a bathochromic shift of the low energy absorption maximum of 7 nm. Related red-shift behavior is also visible for **11**, **12**, and **13**.

**Figure 8:**
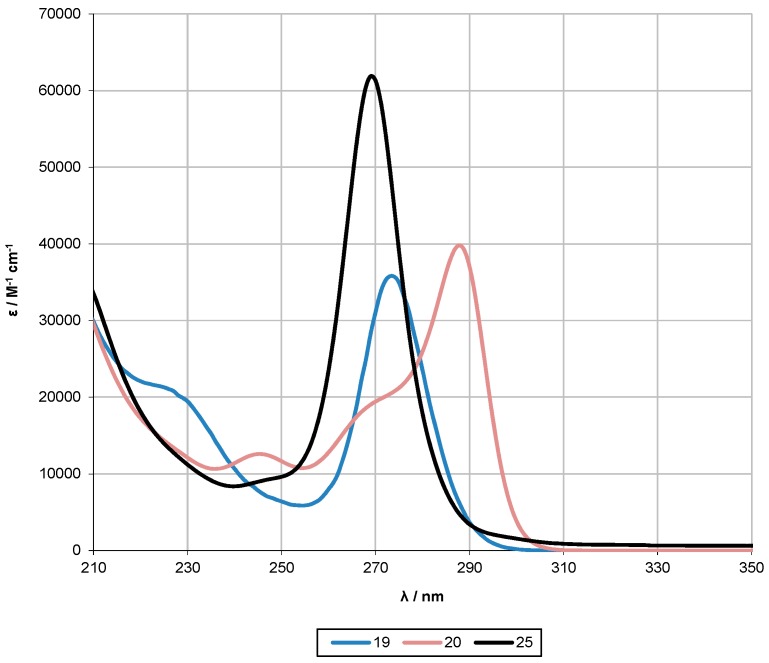
Comparison of the UV spectra of the isomeric compounds **19** and **20** and compound **25**, which can be regarded as the core of compound **19**.

Figure 8 shows the UV spectra of compounds **19**, **20**, and **25**. Compounds **19** and **20** are not only isomers but the main chains of both compounds are nonasilanes. Compound **19** contains two pentamethyldisilanyl units and an internal 1,3-trisilanylene unit, whereas Compound **20** has two tris(trimethylsilyl)silyl units and an internal 1,5-pentasilanylene unit. The UV-spectra of **19** and **20** suggest that the conformations of these two oligosilanes at ambient temperature are quite different. The low energy absorption maximum of **19** is at 273 nm corresponding to an aligned heptasilane fragment. This seems to indicate that the pentamethyldisilanyl units do not participate in the delocalized segment. One reason for this is a likely easy rotation around the Si-Si bonds. Another reason might be that the pentamethyldisilanyl is not inclined to stretch out but to bend back toward the main body of the molecule. Such behavior had been observed by Apeloig and co-workers for the tris(pentamethyldisilanyl)silyl unit and was described as an umbrella effect [30].

Compound **20** on the other hand exhibits two distinct absorption bands at 271 and at 288 nm. The latter can be considered as the low energy absorption maximum of an aligned nonasilane [13] whereas a band around 270 nm hints at a aligned heptasilane segment. The existence of a second conformer in tris(trimethylsilyl)silyl terminated silanes was observed before for 2,2,9,9-tetrakis(trimethylsilyl)octadecamethyldecasilane, where a decasilane band was accompanied by an octasilane band [10]. The reason for the effect of two distinct conformers is that, for spacer lengths of more than four dimethylsilylene units, the steric bulk of the two tris(trimethylsilyl)silyl end groups is not sufficient to align the whole molecule. The UV spectrum of 2,2,6,6-tetrakis(trimethylsilyl)dodecamethylheptasilane (**25**), which is also seen in Figure 8 tells us that for **19** at least some degree of improved alignment is existent as evidence of a slight bathochromic shift of the low energy absorption band from 269 to 273 nm.

The nonasilane band of **20** can also be compared to 2,2,5,5,8,8-hexakis(trimethylsilyl)decamethylnonasilane (**27**) (Table 1) [13], where the two methyl groups at the central silicon atom of the compound are replaced by trimethylsilyl groups. The nonasilane absorption band associated with **27** is only visible as a shoulder and the spectrum is dominated by a prominent hexasilane band [13].

**Figure 9:**
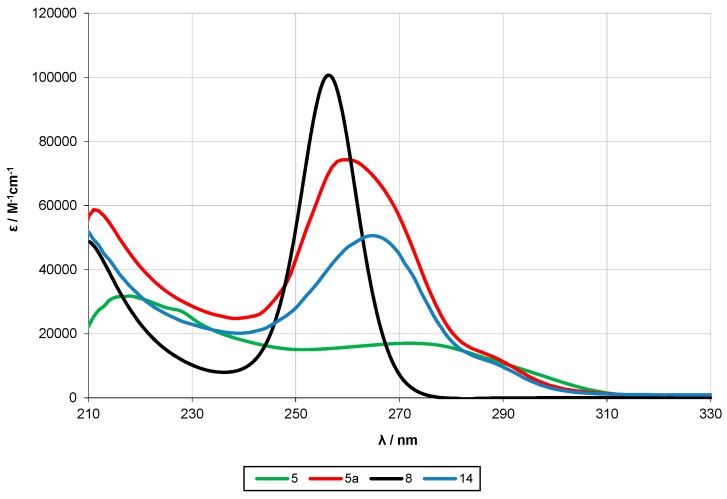
Comparison of the UV spectra of different dodecasilanes (**5**, **5a**, **14**) and compound **8**.

The UV spectra shown in Figure 9 are those of **5**, **5a**, and **14** in addition to compound **8**. We have discussed the difference between **5** and **5a** already above. Compound **14** contains the end-groups of **5** and the core of **5a**. Compared to **5a**, the UV spectrum of **14** shows a bathochromic shift associated with a higher conformational flexibility but the degree of this shift is not as distinct as observed for **5**. This is also emphasized by the shape of the absorption band, which suggests still the presence of a conformational preference. Again, the band of compound **8** tells us that the predominant conformations of **5**, **5a**, and **14** include segments longer or better aligned than the hexasilane unit in **8**.

**Figure 10:**
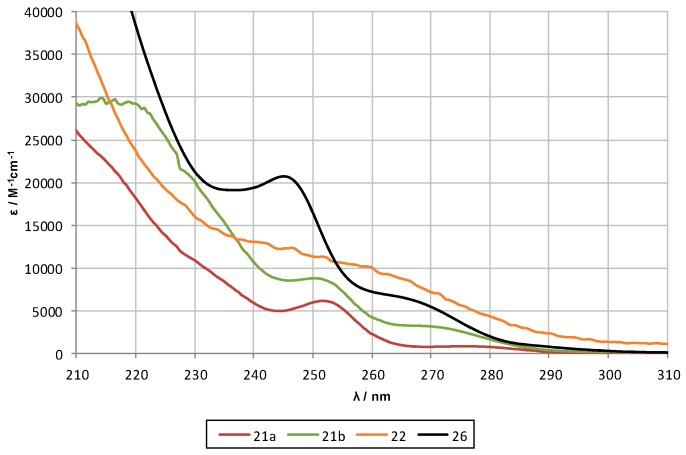
Comparison of the UV spectra of cyclosilanes **21a**, **21b**, **22**, **23**, **24**, and **26**.

The last UV comparison of this study is between cyclosilanes **21a**, **21b**, **22**, and **26** (Figure 10). From seminal work by West and co-workers, it is known that the UV absorption behavior of permethylated cyclosilanes exhibits a trend of hypsochromic shifts for compounds with increasing ring sizes up to the cyclononasilane [31]. Our own studies, however, have shown that UV absorption behavior of trimethylsilyl-substituted cyclosilanes is different from that of permethylated cyclosilanes of the same ring size. Spectroscopic analysis suggested that σ-electron delocalization occurs along chain segments in the ring reaching from one trimethylsilyl group to the other [32]. Using this model for 1,1,4,4,-tetrakis(trimethylsilyl)octamethylcyclohexasilane (**26**) [33,34], a band around 255 nm should be observed. This is indeed true as an absorption maximum is observed at 260 nm (Figure 10).

The spectra for **21a** and **21b** hexasilane bands should be similar but the associated bands are shifted to 251 nm for **21a** and to at 250 nm for **21b**. Both compounds show shoulders that tail into the more bathochromic region. The UV spectrum of **22** is meaningful (Figure 10). Despite some absorption resembling that of **26**, the absorption features are less pronounced. It is not exactly clear whether this absorption behavior is that of the cyclosilane core or includes interaction with its phenyl substituents.

### 2.3. X-ray Crystallography

**Figure 11:**
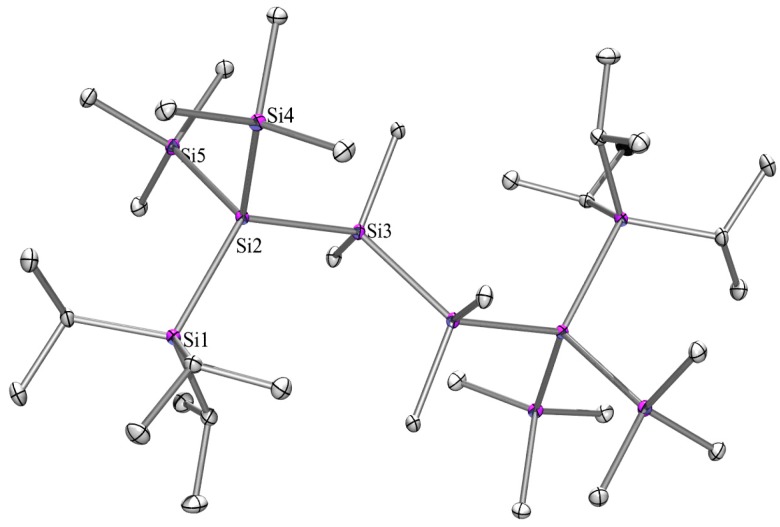
Crystal structure of **9**. Thermal ellipsoids are represented at the 30% level and hydrogen atoms have been omitted for clarity (bond lengths in Å, angles in deg). Si(1)-C(1) 1.9106(17), Si(1)-Si(2) 2.3945(7), Si(2)-Si(4) 2.3764(7), Si(2)-Si(3) 2.4043(9), Si(3)-Si(3A) 2.3943(9), Si(4)-Si(2)-Si(5) 103.71(3), Si(4)-Si(2)-Si(1) 112.86(3), Si(5)-Si(2)-Si(1) 108.66(3), Si(4)-Si(2)-Si(3) 108.10(3), Si(5)-Si(2)-Si(3) 103.58(2), Si(1)-Si(2)-Si(3) 118.52(3), Si(3A)-Si(3)-Si(2) 121.66(3).

**Figure 12:**
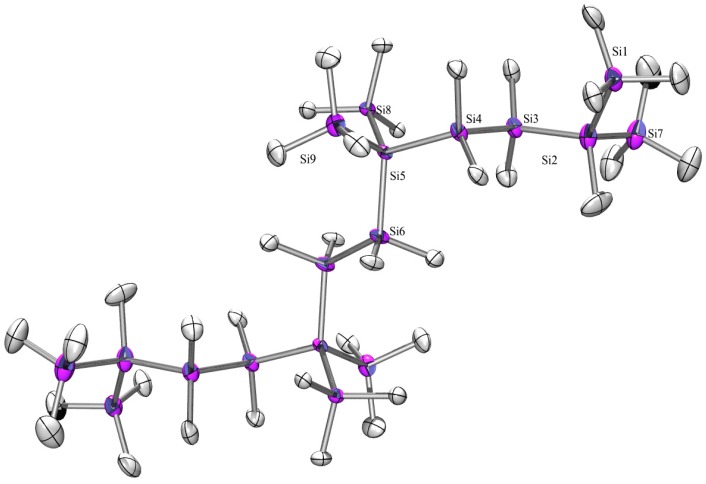
Crystal structure of **14**. Thermal ellipsoids are represented at the 30% level and hydrogen atoms have been omitted for clarity (bond lengths in Å, angles in deg). Si(1)-C(1) 1.875(11), Si(1)-Si(2) 2.358(5), Si(2)-Si(7) 2.333(5), Si(2)-Si(3) 2.357(4), Si(3)-Si(4) 2.342(4), Si(4)-Si(5) 2.379(3), Si(5)-Si(8) 2.354(3), Si(5)-Si(9) 2.371(3), Si(5)-Si(6) 2.376(3), Si(7)-Si(2)-Si(3) 108.05(16), Si(7)-Si(2)-Si(1) 109.16(18), Si(3)-Si(2)-Si(1) 116.28(17), Si(4)-Si(3)-Si(2) 111.55(15), Si(3)-Si(4)-Si(5) 117.83(13), Si(8)-Si(5)-Si(9) 107.50(13), Si(8)-Si(5)-Si(6) 116.91(12), Si(9)-Si(5)-Si(6) 109.33(14), Si(8)-Si(5)-Si(4) 110.18(12), Si(9)-Si(5)-Si(4) 103.60(13), Si(6)-Si(5)-Si(4) 108.51(13).

**Figure 13:**
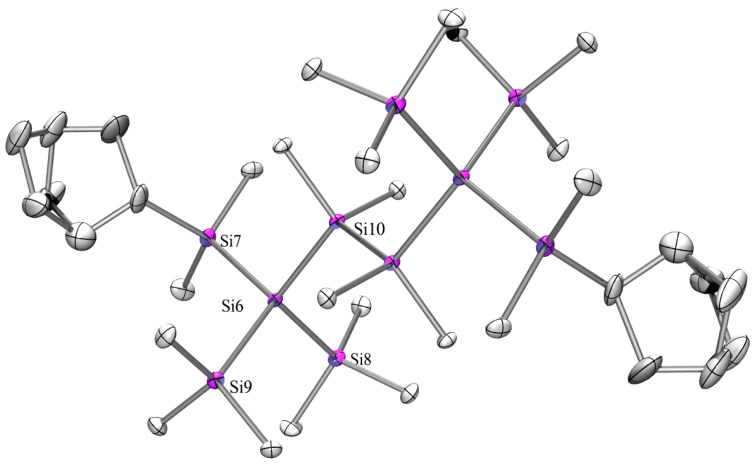
Crystal structure of **15**. Thermal ellipsoids are represented at the 30% level and hydrogen atoms have been omitted for clarity (bond lengths in Å, angles in deg). Si(6)-Si(9) 2.3357(19), Si(6)-Si(8) 2.3460(17), Si(6)-Si(7) 2.357(2), Si(6)-Si(10) 2.3694(17), Si(7)-C(18) 1.869(6), Si(9)-Si(6)-Si(8) 107.60(7), Si(9)-Si(6)-Si(7) 109.89(7), Si(8)-Si(6)-Si(7) 105.08(7), Si(9)-Si(6)-Si(10) 114.57(6), Si(8)-Si(6)-Si(10) 112.08(7), Si(7)-Si(6)-Si(10) 107.21(6).

**Figure 14:**
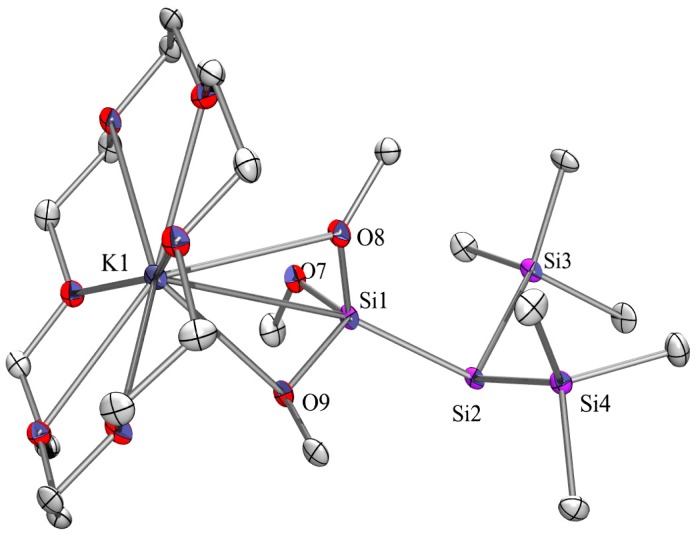
Crystal structure of **16a**. Thermal ellipsoids are represented at the 30% level and hydrogen atoms have been omitted for clarity (bond lengths in Å, angles in deg). K(1)-Si(1) 3.5694(19), Si(2)-Si(1) 2.313(2), Si(2)-Si(3) 2.3386(19), Si(2)-Si(4) 2.350(2), Si(3)-C(18) 1.883(5), Si(1)-O(7) 1.658(4), Si(1)-Si(2)-Si(4) 103.97(7), Si(3)-Si(2)-Si(4) 104.33(7), Si(2)-Si(1)-K(1) 158.14(7).

**Figure 15:**
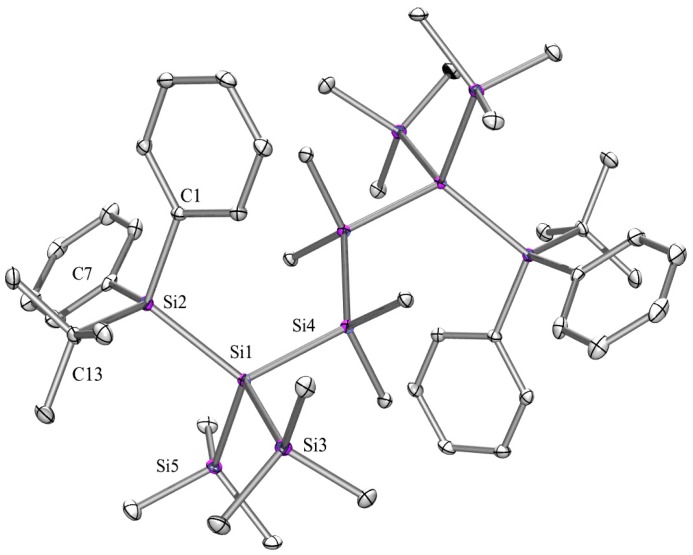
Crystal structure of **17**. Thermal ellipsoids are represented at the 30% level and hydrogen atoms have been omitted for clarity (bond lengths in Å, angles in deg). Si(1)-Si(5) 2.3726(9), Si(1)-Si(3) 2.3806(9), Si(1)-Si(2) 2.4011(8), Si(1)-Si(4) 2.4184(9), Si(2)-C(1) 1.887(2), Si(5)-Si(1)-Si(3) 105.73(3), Si(5)-Si(1)-Si(2) 107.99(3), Si(3)-Si(1)-Si(2) 114.99(3), Si(5)-Si(1)-Si(4) 107.83(3), Si(3)-Si(1)-Si(4) 100.65(3), Si(2)-Si(1)-Si(4) 118.74(3).

**Figure 16:**
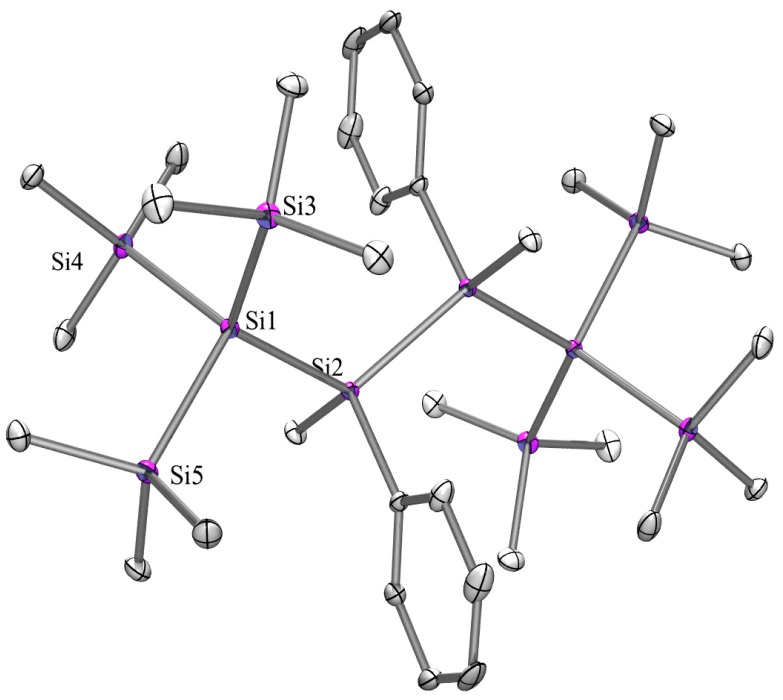
Crystal structure of **18**. Thermal ellipsoids are represented at the 30% level and hydrogen atoms have been omitted for clarity (bond lengths in Å, angles in deg). Si(1)-Si(3) 2.3543(12), Si(1)-Si(4) 2.3592(12), Si(1)-Si(5) 2.3681(13), Si(1)-Si(2) 2.3762(11), Si(2)-C(1) 1.886(3), Si(3)-Si(1)-Si(4) 106.64(4), Si(3)-Si(1)-Si(5) 108.04(4), Si(4)-Si(1)-Si(5) 105.41(5), Si(3)-Si(1)-Si(2) 118.47(4), Si(4)-Si(1)-Si(2) 111.25(4), Si(5)-Si(1)-Si(2) 106.28(4).

**Figure 17:**
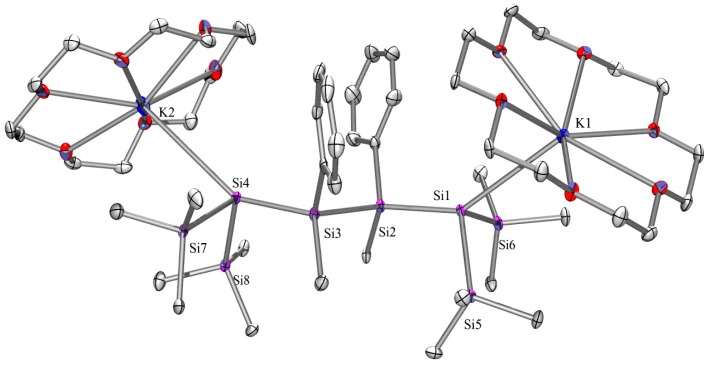
Crystal structure of **18a**. Thermal ellipsoids are represented at the 30% level and hydrogen atoms have been omitted for clarity (bond lengths in Å, angles in deg). Si(1)-Si(5) 2.3449(19), Si(1)-Si(6) 2.350(2), Si(1)-Si(2) 2.360(2), Si(1)-K(1) 3.5079(19), Si(2)-C(1) 1.908(5), Si(2)-Si(3) 2.3734(17), Si(5)-Si(1)-Si(6) 99.17(7), Si(5)-Si(1)-Si(2) 106.41(7), Si(6)-Si(1)-Si(2) 98.25(7), Si(1)-Si(2)-Si(3) 117.41(7), Si(2)-Si(3)-Si(4) 115.99(7), Si(7)-Si(4)-Si(8) 96.39(7), Si(7)-Si(4)-Si(3) 99.23(8), Si(8)-Si(4)-Si(3) 104.83(7).

**Figure 18:**
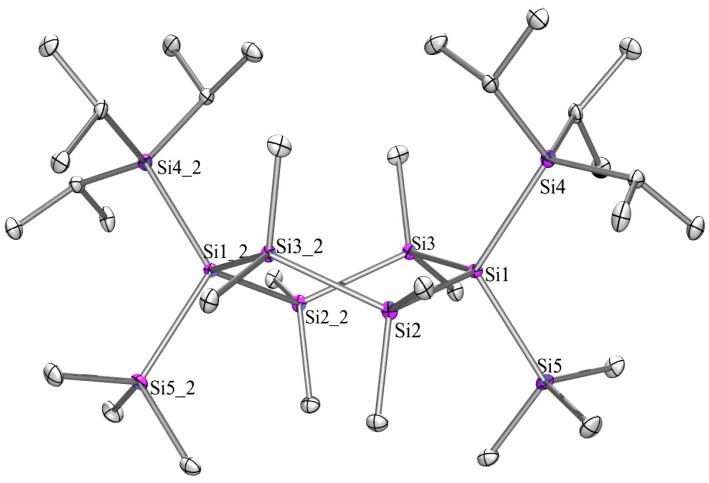
Crystal structure of **21a**. Thermal ellipsoids are represented at the 30% level and hydrogen atoms have been omitted for clarity (bond lengths in Å, angles in deg). Si(1)-Si(5) 2.380(2), Si(1)-Si(3) 2.381(2), Si(1)-Si(2) 2.382(2), Si(1)-Si(4) 2.408(2), Si(2)-C(2) 1.882(6), Si(5)-Si(1)-Si(3) 105.57(7), Si(5)-Si(1)-Si(2) 104.19(8), Si(3)-Si(1)-Si(2) 106.93(7), Si(5)-Si(1)-Si(4) 113.14(8), Si(3)-Si(1)-Si(4) 112.16(8), Si(2)-Si(1)-Si(4) 114.13(8).

**Figure 19:**
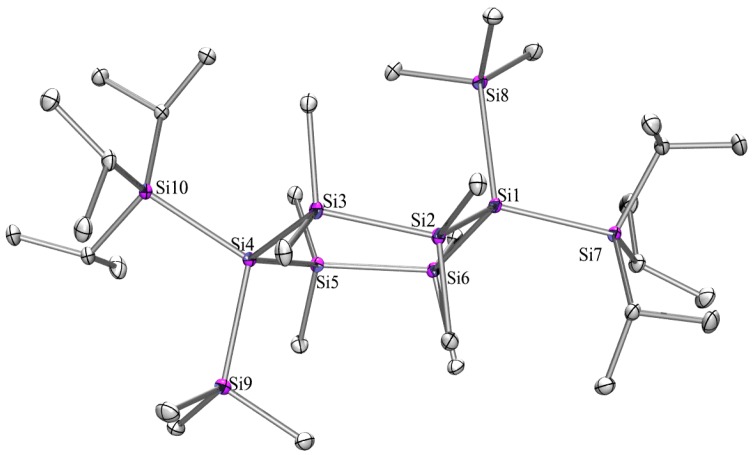
Crystal structure of **21b**. Thermal ellipsoids are represented at the 30% level and hydrogen atoms have been omitted for clarity (bond lengths in Å, angles in deg). Si(1)-Si(2) 2.3613(7), Si(1)-Si(6) 2.3714(7), Si(1)-Si(8) 2.3734(8), Si(1)-Si(7) 2.4033(7), Si(2)-C(1) 1.8900(17), Si(2)-Si(3) 2.3603(7), Si(3)-Si(4) 2.3842(7), Si(4)-Si(5) 2.3899(8), Si(4)-Si(9) 2.3987(7), Si(4)-Si(10) 2.4169(8), Si(5)-Si(6) 2.3957(7), Si(2)-Si(1)-Si(6) 102.27(3), Si(2)-Si(1)-Si(8) 110.43(3), Si(6)-Si(1)-Si(8) 106.77(3), Si(2)-Si(1)-Si(7) 110.93(3), Si(6)-Si(1)-Si(7) 115.81(4), Si(8)-Si(1)-Si(7) 110.29(3), Si(3)-Si(2)-Si(1) 117.55(3), Si(2)-Si(3)-Si(4) 117.96(4), Si(3)-Si(4)-Si(5) 109.23(3), Si(3)-Si(4)-Si(9) 109.41(3), Si(5)-Si(4)-Si(9) 103.01(3), Si(3)-Si(4)-Si(10) 110.47(4), Si(5)-Si(4)-Si(10) 114.69(3), Si(9)-Si(4)-Si(10) 109.71(3), Si(4)-Si(5)-Si(6) 120.74(3), Si(1)-Si(6)-Si(5) 119.81(3).

**Figure 20:**
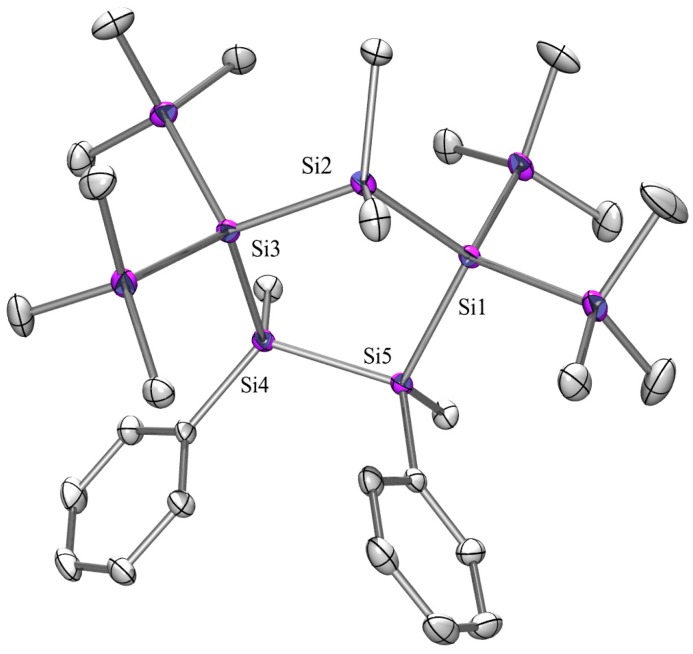
Crystal structure of **22**. Thermal ellipsoids are represented at the 30% level and hydrogen atoms have been omitted for clarity (bond lengths in Å, angles in deg). Si(1)-Si(5) 2.3632(8), Si(1)-Si(7) 2.3643(10), Si(1)-Si(2) 2.3684(10), Si(1)-Si(6) 2.3696(9), Si(2)-Si(3) 2.3796(10), Si(3)-Si(9) 2.3617(8), Si(3)-Si(8) 2.3637(9), Si(3)-Si(4) 2.3667(9), Si(4)-Si(5) 2.3570(9), Si(5)-Si(1)-Si(7) 107.82(4), Si(5)-Si(1)-Si(2) 104.09(3), Si(7)-Si(1)-Si(2) 112.73(3), Si(5)-Si(1)-Si(6) 112.97(3), Si(7)-Si(1)-Si(6) 105.35(3), Si(2)-Si(1)-Si(6) 113.90(3), Si(1)-Si(2)-Si(3) 107.73(3), Si(9)-Si(3)-Si(8) 106.82(3), Si(9)-Si(3)-Si(4) 107.26(3), Si(8)-Si(3)-Si(4) 113.59(3), Si(9)-Si(3)-Si(2) 111.69(3), Si(8)-Si(3)-Si(2) 112.20(3), Si(4)-Si(3)-Si(2) 105.27(3), Si(5)-Si(4)-Si(3) 107.18(4), Si(4)-Si(5)-Si(1) 104.61(3).

**Figure 21:**
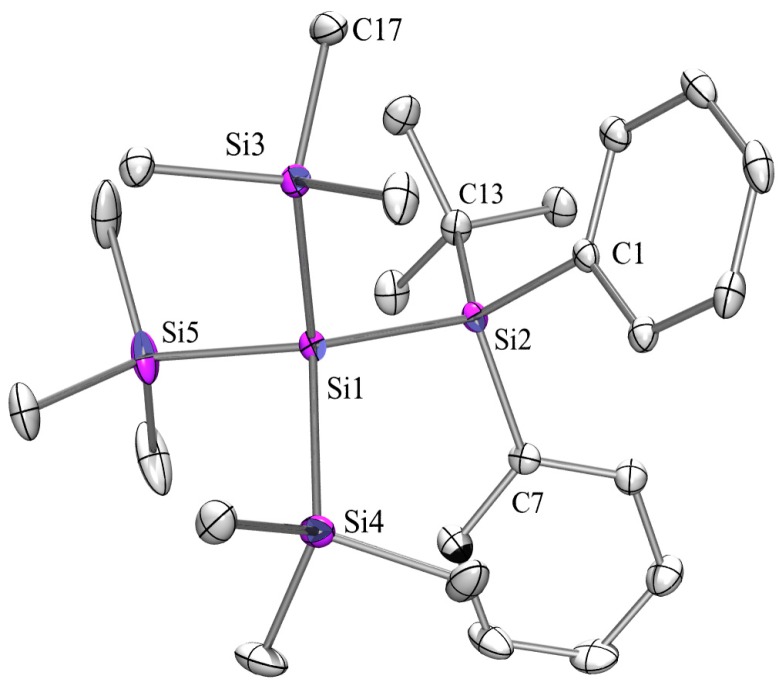
Crystal structure of **23**. Thermal ellipsoids are represented at the 30% level and hydrogen atoms have been omitted for clarity (bond lengths in Å, angles in deg). Si(1)-Si(3) 2.3600(10), Si(1)-Si(5) 2.3654(10), Si(1)-Si(4) 2.3727(10), Si(1)-Si(2) 2.3827(10), Si(3)-Si(1)-Si(5) 107.02(4), Si(3)-Si(1)-Si(4) 105.31(4), Si(5)-Si(1)-Si(4) 103.48(4), Si(3)-Si(1)-Si(2) 108.01(3), Si(5)-Si(1)-Si(2) 119.85(3), Si(4)-Si(1)-Si(2) 112.18(3).

**Figure 22:**
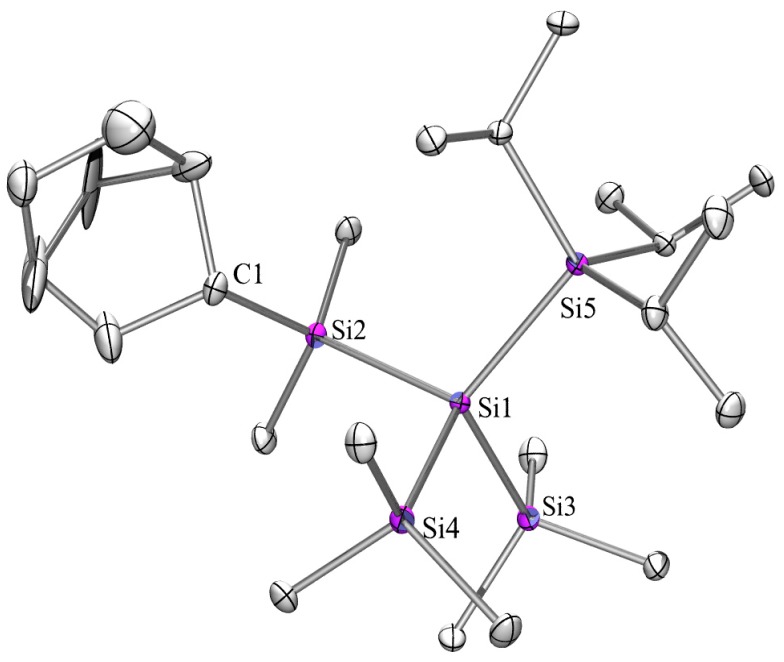
Crystal structure of **24**. Thermal ellipsoids are represented at the 30% level and hydrogen atoms have been omitted for clarity (bond lengths in Å, angles in deg). C(1)-C(2) 1.543(5), C(1)-Si(2) 1.914(3), Si(1)-Si(4) 2.3649(11), Si(4)-Si(1)-Si(3) 104.76(4), Si(4)-Si(1)-Si(2) 106.26(4), Si(3)-Si(1)-Si(2) 105.44(4).

Compounds **9** (Figure 11), **14** (Figure 12), **15** (Figure 13), **16a** (Figure 14), **17** (Figure 15), **18** (Figure 16), **18a** (Figure 17), **21a** (Figure 18), **21b** (Figure 19), **22** (Figure 20), **23** (Figure 21), and **24** (Figure 22) of this study were characterized by X-ray single-crystal structure analysis (for tables concerning crystallographic information see: Supplementary Information). As numerous related polysilanes structures are already known, these compounds provide an excellent opportunity to compare structural properties of organooligosilanes.

Compounds **9**, **14**, **18**, **15**, and **17** are oligosilanes with longer chains and their structure in the solid state can provide insight into the effectiveness of σ-electron delocalization.

The structures of compounds with a 1,2-disilanylene spacer between the tris(trimethylsilyl)silyl groups were found to crystallize in monoclinic space groups for **9** and **18** and the triclinic space group P-1 for **15** and **17**. For all compounds, an inversion center between the two central silicon atoms was found. The asymmetric units of **15** and **17** are containing only two half molecules. The structures of three other compounds with a 1,2-disilanylene spacers between the tris(trimethylsilyl)silyl or -germyl groups were reported to be triclinic (P-1), namely 1,1,1,4,4,4-hexakis(trimethylsilyl)-2,2,3,3-tetramethyltetrasilane [35], 1,2-bis[tris(trimethylsilyl)germyl]tetramethyldisilane [36], and 1,2-bis[tris(trimethylsilyl)germyl]tetramethyldigermane [36].

The longer polysilane **14** also crystallizes in the triclinic space group P-1 again with an inversion center in the silicon backbone. Its main chain is divided into three segments which was observed earlier with similar structures [13]. After the bis(trimethylsilyl)silylene unit, the skeleton makes a turn adopting a torsional angle of 83°. Table 2 reports the conformational properties of the oligosilanes by listing the torsional angles along the main chain. The conformations of **9**, **18**, **15**, and **17** can be described as *transoid-anti-transoid* (TAT) [2].

It should be noted that the first torsional angle of **9** is with 168° more *transoid* than those of **18**, **15** and **17**. Given the fact that **9** exhibits the most bathochromicly shifted absorption band of the structurally related permethylated compounds, this might be in part caused by a more effective delocalization. The structure of **14** is particularly interesting. Judging the torsional angles, an ATOTATOTA conformation can be assigned. Compared to the structure of **5a** [13], which has tris(trimethylsilyl)silyl end groups, **14** features more aligned chain end segments.

For 1,4-substituted cyclohexasilanes, a chair conformation with the large substituents in equatorial positions is typical [14,33,38,39,40]. However, 1,1,4,4,-tetrakis(trimethylsilyl)octamethylcyclohexasilane (**26**) crystallizing in the monoclinic space group C2/c is an exception as all four trimethylsilyl groups have the same steric demand allowing the system to adopt a twist conformation [34]. Changing two trimethylsilyl against triisopropylsilyl groups as in **21** led to two isomers whose crystals differ in shape so that they can be separated under the microscope. Both isomers crystallize in the space group C2/c. In the *cis*-isomer (**21a**) the two triisopropylsilyl groups occupy axial positions and the ring adopts a twist conformation whereas in **21b** the substituents are both in equatorial position and the ring exhibits a chair conformation. A chair conformation was also reported for the related 1,4-bis(*tert*-butyldimethylsilyl)-1,4-bis(trimethylsilyl)octamethylcyclohexasilane with the two *tert*-butyldimethylsilyl groups in equatorial positions [33]. The cyclopentasilane ring in **22** exhibits an envelope conformation with one MePhSi unit on the flap and the phenyl groups in *cis* orientation.

The two neopentasilanes **23** and **24** crystallize both in the monoclinic space group P2(1)/c. The structures are unexceptional with the Si-Si distances of 2.340 Å in tetrakis(trimethylsilyl)silane [41] being elongated to 2.364–2.385 Å in **24** and 2.360–2.383 Å in **23**. 

The structures of the silyl anions **16a** and **18a** show both unexpected motives. While it is typical that the two potassium atoms of the silandiide **18a** are coordinated by 18-crown-6 ethers, a torsional angle of 48.0° for K1-Si1-Si4-K2, which leads to a *gauche* conformation is rather unusual. For the few known structures of other crown ether potassium silyl dianions [33,42,43], the respective torsion angles for K-Si-Si-K are ranging from 180.0° to 147.7° and therefore consequently show *trans* conformation. The only exception with a smaller angle was 1,1,5,5-tetrakis(trimethylsilyl)hexamethylpentasilanyl-1,5-dipotassium [42] with an *ortho* conformation (84.8°).

Typically, the position of the potassium atom in solid state structures of potassium silanides is that of a tetrahedral substituent. Recently, some 18-crown-6 potassium oligosilanylsilatranes were published which show a distortion of the potassium atom in order to coordinate to both the anionic silicon atom and to an oxygen atom of remaining molecular scaffold [44]. In the silanides (MeOMe_2_Si)_3_SiK [45] and (MeOCH_2_CH_2_OMe_2_Si)_3_SiK [46], the potassium atom coordinates solely to the alkoxy groups. In **16a,** a similar pattern can be observed where the potassium is coordinating exclusively to two oxygen atoms of the (MeO)_3_Si substituent.

## 3. Experimental Section

*General Remarks:* All reactions involving air-sensitive compounds were carried out under an atmosphere of dry nitrogen or argon using either Schlenk techniques or a glove box. Solvents were dried using a column solvent purification system [47]. All chemicals were obtained from different suppliers and were used without further purification. 1,2-Dichlorotetramethyldisilane [48,49], 1,3-dichlorohexamethyltrisilane [48,49], 1,4-dichlorooctamethyltetrasilane [48,49], tetrakis(trimethylsilyl)silane [50], isopropylbis(trimethylsilyl)silylpotassium [19], 2,5-bis(trimethylsilyl)dodecamethylhexasilane (**3**) [51], bis(trimethylsilyl)triisopropylsilylsilylpotassium [19], tris(trimethylsilyl)triisopropylsilylsilane [19], 2,2,5-tris(trimethylsilyl)undecamethylhexasilane [21], 2,2,6,6-tetrakis(trimethylsilyl)dodecamethylheptasilane (**25**) [52], 2,2,5-tris(trimethylsilyl)undecamethylhexasilane [21], tris(trimethylsilyl)]trimethoxysilylsilane [53], 1,2-dimethyltetraphenyldisilane [54] and 1,1,4,4,-tetrakis(trimethylsilyl)octamethylcyclohexasilane (**26**) [33] were prepared according to literature procedures.

^1^H- (300 MHz), ^13^C- (75.4 MHz), and ^29^Si- (59.3 MHz) NMR spectra were recorded on a Varian Unity INOVA 300 (Varian, Palo Alto, CA, USA). Samples for ^29^Si spectra were either dissolved in deuterated solvents or in cases of reaction samples measured with a D_2_O capillary in order to provide an external lock frequency signal. To compensate for the low isotopic abundance of ^29^Si, the INEPT pulse sequence [55,56] was used for the amplification of the signal. If not noted, otherwise the used solvent was C_6_D_6_ and samples were measured at rt. Elementary analysis was carried using a Heraeus VARIO ELEMENTAR EL apparatus (Hanau, Germany). UV spectra were measured on a Perkin Elmer Lambda 35 spectrometer (Perkin-Elmer Corp., Waltham, MA, USA) using spectroscopy grade pentane as solvent.

For X-ray structure analyses, the crystals were mounted onto the tip of glass fibers, and data collection was performed with a BRUKER-AXS SMART APEX CCD diffractometer (Bruker-AXS: Madison, WI, USA) using graphite-monochromated Mo K_α_ radiation (0.71073 Å). The data were reduced to F_o_^2^ and corrected for absorption effects with SAINT [57] and SADABS [58,59], respectively. Structures were solved by direct methods and refined by full-matrix least-squares method (SHELXL97 and SHELX2013) [60]. All non-hydrogen atoms were refined with anisotropic displacement parameters. Hydrogen atoms were placed in calculated positions to correspond to standard bond lengths and angles. Crystallographic data (excluding structure factors) for the structures of compounds **9**, **14**, **15**, **16a**, **17**, **18**, **18a**, **21a**, **21b**, **22**, **23** and **24** reported in this paper have been deposited with the Cambridge Crystallographic Data Center as supplementary publication no. CCDC 670864 (**9**), 975429 (**14**), 1486947 (**15**), 1486948 (**16a**), 1486950 (**17**), 1486951 (**18**), 1027155 (**18a**), 974732 (**21a**), 974731 (**21b**), 1486946 (**22**), 1486949 (**23**), and 1486945 (**24**). Copies of data can be obtained free of charge at: http://www.ccdc.cam.ac.uk/products/csd/request/. Figures of solid state molecular structures were generated using Ortep-3 as implemented in WINGX [61] and rendered with POV-Ray 3.6 [62].

*Bis(trimethylsilyl)diisopropylsilane* (**1**). A solution of isopropylbis(trimethylsilyl)silyl potassium (1.272 g, 2.431 mmol) in benzene (20 mL) was added dropwise at 0 °C to a solution of 2-chloropropane (0.191 g, 1.00 equiv) in benzene (10 mL). The reaction mixture remained colorless during the whole addition process and a white precipitate was formed. After 2 h at 0 °C, the suspension was allowed to come to rt followed by an aqueous workup (diethyl ether). Compound **1** (484 mg, 74%) was obtained as a colorless liquid. ^1^H-NMR (δ in ppm): 1.18 (m, 2H, CH), 1.11 (d, *J* = 6.3 Hz, 12H, C*H*_3_CH), 0.20 (s, 18H, Me_3_Si). ^13^C-NMR (δ in ppm): 21.2 (*C*H_3_CH), 12.5 (CH), 1.4 (*Me_3_*Si). ^29^Si-NMR (δ in ppm): −17.0 (Me_3_Si), −27.4 (Me_3_Si*Si*). MS (70 eV) *m*/*z* (%): 260 (33.7) [M^+^], 245 (6.5) [M^+^ − Me], 217 (27.4) [M^+^ − CHMe_2_], 175 (61.5) [M^+^ − (CHMe_2_)_2_], 73 (100) [SiMe_3_^+^].

*2,2,5,5-Tetraisopropyldecamethylhexasilane* (**2**). Same procedure as described for **1** using 1,2-dichlorotetramethyldisilane (54 mg, 0.289 mmol) and for the preparation of the silanide: **1** (150 mg, 0.577 mmol), 18-crown-6 (152 mg, 1.00 equiv), and KO*^t^*Bu (65 mg, 1.00 equiv) (^29^Si-NMR (δ in ppm, D_2_O capillary): −14.4 (Me_3_*Si*SiK)), −54.0 (Me_3_Si*Si*K)). Compound **2** (360 mg, 90%) was obtained as a colorless crystalline solid. Mp: 199–206 °C. ^1^H-NMR (δ in ppm): 1.33 (m, 4H, CH), 1.18 (d, *J* = 7.8 Hz, 12H, CH_3_), 1.15 (d, *J* = 7.8 Hz, 12H, CH_3_), 0.47 (s, 12H, Me_2_Si), 0.28 (s, 18H, Me_3_Si). ^13^C-NMR (δ in ppm): 21.7 (CH_3_), 21.5 (CH_3_), 12.0 (CH), 2.2 (Me_3_Si), −0.3 (Me_2_Si). ^29^Si-NMR (δ in ppm): −15.7 (Me_3_Si), −18.3 (Me_3_Si*Si*), −38.6 (Me_2_Si). MS (70 eV) *m*/*z* (%): 490 (4.0) [M^+^], 303 (9.4) [M^+^ − Si_2_Me_3_(CH)_2_(CH_3_)_4_], 245 (26.3) [M^+^ − Si_2_Me_4_(CH)_3_(CH_3_)_6_], 203 (44.2) [Si_4_Me_6_H^+^], 187 (24.8) [Si_4_Me_5_^+^], 172 (57.8) [Si_4_Me_4_^+^], 73 (100) [SiMe_3_^+^]. Anal. calcd. for C_22_H_58_Si_6_ (491.22): C 53.79, H 11.90. Found: C 53.42, H 11.85. UV absorption: λ_1_ = 209 nm (ε_1_ = 1.7 × 10^4^ M^−1^·cm^−1^), λ_2_ = 260 nm (ε_2_ = 2.4 × 10^4^ M^−1^·cm^−1^), shoulder: 227 nm.

*2,5,8,11-Tetrakis(trimethylsilyl)docosamethyldodecasilane* (**5**). To a solution of 1,2-dichlorotetramethyldisilane (38 mg, 0.202 mmol) in THF (30 mL) at 0 °C a solution of 5-trimethylsilyldodecamethylhexasilanyl-2-potassium (**4**) (freshly prepared from: 2,5-bis(trimethylsilyl)dodecamethylhexasilane (**3**) (200 mg, 0.404 mmol), KO*^t^*Bu (48 mg, 0.424 mmol) in THF (20 mL); **4**: ^29^Si-NMR (δ in ppm, D_2_O capillary): −6.8 (Me_3_*Si*SiK), −12.5 (Me_3_*Si*SiMe), −26.5 (Me_2_Si), −35.7 (Me_2_Si), −88.1 (MeSi), −129.5 (MeSiK)) was added dropwise. After 14 h stirring at rt it was poured into a mixture of H_2_SO_4_ (0.5 M), diethylether, and ice. The layers were separated and the organic layer dried with Na_2_SO_4_. After removal of the solvent the two isomers of **5** were obtained as a colorless oil (136 mg, 70%). ^1^H-NMR (δ in ppm, CDCl_3_): 0.31–0.23 (m, 36H), 0.18 (s, 36H), 0.16 (s, 12H), 0.13 (s, 18H). ^13^C-NMR (δ in ppm, CDCl_3_): 1.4, 1.0, 0.4, −1.0, −1.1, −1.1, −1.2, −1.2, −1.5, −1.6, −9.3, −11.4. ^29^Si-NMR (δ in ppm, CDCl_3_): −11.6 (Me_3_Si), −11.7 (Me_3_Si), −11.8 (Me_3_Si), −11.8 (Me_3_Si), −33.1 (Me_2_Si), −33.7 (Me_2_Si), −33.7 (Me_2_Si), −33.9 (Me_2_Si), −34.0 (Me_2_Si), −73.8 (MeSi), −74.3 (MeSi), −81.6 (MeSi). UV absorption: λ_1_ = 218 nm (ε_1_ = 3.2 × 10^4^ M^−1^·cm^−1^), λ_2_ = 268 nm (ε_2_ = 1.6 × 10^4^ M^−1^·cm^−1^).

*2,5,9,12-Tetrakis(trimethylsilyl)tetracosamethyltridecasilane* (**6**). Same procedure as described for **5** using: **4** (0.404 mmol) and 1,3-dichlorohexamethyltrisilane (0.202 mmol, 50 mg). Compound **6** was obtained as colorless oil (191 mg, 93%, mixture of both isomers). ^1^H-NMR (δ in ppm, CDCl_3_): 0.30–0.22 (m, 36H), 0.18 (s, 36H), 0.16 (s, 12H), 0.13 (s, 24H). ^13^C-NMR (δ in ppm, CDCl_3_): 1.4, 1.0, 0.4, −1.1, −1.2, −1.3, −1.5, −1.6, −2.9, −9.4, −11.4. ^29^Si-NMR (δ in ppm, CDCl_3_): −11.7 (Me_3_Si), −11.8 (Me_3_Si), −11.9 (Me_3_Si), −33.2 (Me_2_Si), −33.7 (Me_2_Si), −33.8 (Me_2_Si), −34.0 (Me_2_Si), −34.2 (Me_2_Si), −34.7 (Me_2_Si), −36.3 (Me_2_Si), −36.4 (Me_2_Si), −74.4 (MeSi), −75.3 (MeSi), −81.6 (MeSi), −82.0 (MeSi). UV absorption: λ_1_ = 212 nm (ε_1_ = 2.7 × 10^4^ M^−1^·cm^−1^), λ_2_ = 272 nm (ε_2_ = 1.7 × 10^4^ M^−1^·cm^−1^), shoulder: 287 nm.

*2,5,10,13-Tetrakis(trimethylsilyl)hexacosamethyltetradecasilane* (**7**). Same procedure as described for **5** using: **4** (0.404 mmol) and 1,4-dichlorooctamethyltetrasilane (0.202 mmol, 62 mg). Compound **7** was obtained as colorless oil (193 mg, 89%, mixture of both isomers). ^1^H-NMR (δ in ppm, CDCl_3_): 0.29–0.22 (m, 36H), 0.18 (s, 36H), 0.16 (s, 12H), 0.13 (s, 18H), 0.13 (s, 12H). ^13^C-NMR (δ in ppm, CDCl_3_): 1.4, 1.0, 0.5, −1.1, −1.2, −1.3, −1.5, −1.6, −3.2, −3.2, −9.5, −11.4. ^29^Si-NMR (δ in ppm, CDCl_3_): 11.7 (Me_3_Si), −11.8 (Me_3_Si), −11.9 (Me_3_Si), −33.7 (Me_2_Si), −33.8 (Me_2_Si), −34.0 (Me_2_Si), −36.2 (Me_2_Si), −36.4 (Me_2_Si), −74.5 (MeSi), −75.5 (MeSi), −81.6 (MeSi), −82.0 (MeSi). UV absorption: λ_1_ = 222 nm (ε_1_ = 3.6 × 10^4^ M^−1^·cm^−1^), λ_2_ = 285 nm (ε_2_ = 2.4 × 10^4^ M^−1^·cm^−1^).

*2,5-Bis(triisopropylsilyl)-2,5-bis(trimethylsilyl)decamethylhexasilan* (**9**). To a solution of 1,2-dichlorotetramethyldisilane (1.30 mmol, 243 mg) in THF (20 mL) a solution of bis(trimethylsilyl)triisopropylsilylsilyl potassium (2.47 mmol, 916 mg) in THF (10 mL) was added dropwise. After 6 h same the work up procedure as for **5** was applied and crystallization from pentane/acetone yielded colorless crystalline **9** (908 mg, 91%). Mp.: 221–225 °C. ^1^H-NMR (δ in ppm, CDCl_3_): 1.32 (sept, 6H, *J* = 7 Hz, C*H*(CH_3_)_2_), 1.22 (d, 36H, *J* = 7 Hz, CH(C*H_3_*)*_2_*), 0.50 (s, 12H, SiMe_2_), 0.33 (s, 36H, SiMe_3_). ^13^C-NMR (δ in ppm, CDCl_3_): 20.6 (CH(*C*H_3_)_2_), 15.2 (Si*C*H(CH_3_)_2_), 5.0 (SiMe_3_), 3.1 (SiMe_2_). ^29^Si-NMR (δ in ppm, CDCl_3_): 11.1 (*^i^*Pr_3_Si), −9.1 (Me_3_Si), −24.5 (Me_2_Si), −120.3 (Me_3_Si*Si*). Anal. calcd. for C_34_H_90_Si_10_ (779.94): C 52.36, H 11.63. Found: C 52.09, H 11.58. UV Absorption: λ_1_ = 217 nm (ε_1_ = 2.7 × 10^4^ M^−1^·cm^−1^), λ_2_ = 264 nm (ε_2_ = 1.4 × 10^4^ M^−1^·cm^−1^), shoulder: 230 nm.

*2,5,8,11-Tetrakis(triisopropylsilyl)-2,5,8,11-tetrakis(trimethylsilyl)docosamethyl-dodeca-silane* (**11**). Same procedure as described for **5** using: 2,5-bis(triisopropylsilyl)-5-tris(trimethylsilyl)decamethylhexasilyl-2-potassium (**10**) (freshly prepared from **9** (200 mg, 0.256 mmol) and KO*^t^*Bu (30 mg, 0.269 mmol) in THF (10 mL)) **10**: ^1^H-NMR (δ in ppm): 1.42 (m, 36H), 1.34 (s, 12H), 1.32 (s, 6H), 0.83 (s, 6H), 0.67 (s, 6H), 0.55 (s, 12H), 0.53 (s, 18H). ^29^Si-NMR (δ in ppm): 22.4 (*^i^*Pr_3_*Si*SiK), 13.5 (*^i^*Pr_3_*Si*Si_q_), −7.2 (Me_3_*Si*SiK), −9.6 (Me_3_Si), −14.2 (Me_2_*Si*SiK), −35.2 (Me_2_Si), −126.0 (Si_q_), −189.7 (SiK).] and 1,2-dichlorotetramethyldisilane (0.202 mmol, 62 mg). After 3 h same work up procedure as for **5** yielded colorless crystalline **11** (220 mg, 87%, mixture of both isomers). ^1^H-NMR (δ in ppm): 1.10–1.37 (m, 50H), 0.43 (s, 36H), 0.33 (s, 18H), 0.31 (s, 36H). ^29^Si-NMR (δ in ppm): 15.9 (*^i^*Pr_3_Si), 15.9 (*^i^*Pr_3_Si), 13.9 (*^i^*Pr_3_*Si*), 13.5 (*^i^*Pr_3_*Si*), −9.1 (Me_3_Si), −9.2 (Me_3_Si), −9.2 (Me_3_Si), −9.3 (Me_3_Si), −9.4 (Me_3_Si), −24.1 (Me_2_Si), −24.5 (Me_2_Si), −28.2 (Me_2_Si), −32.0 (Me_2_Si), −113.2 (Si_q_), −120.0 (Si_q_), −125.0 (Si_q_), −125.2 (Si_q_). UV absorption: λ_1_ = 216 nm (ε_1_ = 3.1 × 10^4^ M^−1^·cm^−1^), λ_2_ = 264 nm (ε_2_ = 5.1 × 10^4^ M^−1^·cm^−1^), shoulder: 232 nm.

*2,5,9,12-Tetrakis(triisopropylsilyl)-2,5,9,12-tetrakis(triisopropylsilyl)tetracosamethyl-tri-deca-silane* (**12**). Same procedure as described for **11** using: **10** (0.152 mmol) and 1,3-dichlorhexamethyltrisilane (40 mg, 0.161 mmol). After 5 h same work up procedure as for **5** yielded **12** colorless oil (271 mg, 71%, mixture of both isomers). ^1^H-NMR (δ in ppm): 1.10–1.39 (m, 50H), 0.48 (s, 42H), 0.32 (s, 18H), 0.30 (s, 36H). ^29^Si-NMR (δ in ppm): 16.0 (*^i^*Pr_3_*Si*), 14.1 (*^i^*Pr_3_*Si*), 13.6 (*^i^*Pr_3_*Si*), −9.1 (Me_3_*Si*), −9.2 (Me_3_*Si*), −9.3 (Me_3_*Si*), −9.4 (Me_3_*Si*), −9.4 (Me_3_*Si*), −10.7 (Me_3_*Si*), −23.7 (Me_2_*Si*), −24.2 (Me_2_*Si*), −28.1 (Me_2_*Si*), −32.0 (Me_2_*Si*), −32,7 (Me_2_*Si*), −32.8 (Me_2_*Si*), −33.0 (Me_2_*Si*), −33.0 (Me_2_*Si*), −108.3 (*Si_q_*), −119.7 (*Si_q_*), −124.9 (*Si_q_*), 125.1 (*Si_q_*). UV absorption: λ_1_ = 219 nm (ε_1_ = 2.8 × 10^4^ M^−1^·cm^−1^), λ_2_ = 267 nm (ε_2_ = 4.6 × 10^4^ M^−1^·cm^−1^), λ_3_ = 283 nm (shoulder), λ_4_ = 307 nm (shoulder).

*2,5,10,13-Tetrakis(triisopropylsilyl)-2,5,10,13-tetrakis(triisopropylsilyl)hexacosamethyl-tetra-decasilane* (**13**). Same procedure as described for **11** using: **10** (0.152 mmol) and 1,4-dichlorooctamethyltetrasilane (51 mg, 0.167 mmol). After 5 h same work up procedure as for **5** yielded **13** as a colorless oil (211 mg, 81%, mixture of both isomers). ^1^H-NMR (δ in ppm): 1.12–1.37 (m, 50H), 0.48 (s, 48H), 0.32 (s, 18H), 0.30 (s, 36H). ^29^Si-NMR (δ in ppm): 16.9 (*^i^*Pr_3_Si), 14.2 (*^i^*Pr_3_Si), 14.1 (*^i^*Pr_3_Si), 11.1 (*^i^*Pr_3_Si), −9.1 (Me_3_Si), −9.2 (Me_3_Si), −9.3 (Me_3_Si), −9.3 (Me_3_Si), −23.1 (Me_2_Si), −24.1 (Me_2_Si), −28.8 (Me_2_Si), −29.2 (Me_2_Si), −29.5 (Me_2_Si), −29.6 (Me_2_Si), −31.3 (Me_2_Si), −32.0 (Me_2_Si), −104.6 (Si_q_), −106.9 (Si_q_), −119.3 (Si_q_), −119.4 (Si_q_). UV absorption: λ_1_ = 220 nm (ε_1_ = 2.9 × 10^4^ M^−1^·cm^−1^), λ_2_ = 268 nm (ε_2_ = 4.5 × 10^4^ M^−1^·cm^−1^), λ_3_ = 293 nm (ε_2_ = 3.8 × 10^4^ M^−1^·cm^−1^), λ_4_ = 311 nm (shoulder).

*2,5,8,11-Tetrakis(trimethylsilyl)icosamethyldodecasilan* (**14**). Same procedure as described for **5** using 2,5-bis(trimethylsilyl)undecamethylhexasilanyl-2-potassium (1.63 mmol) (prepared from: 2,2,5-tris(trimethylsilyl)undecamethylhexasilane (900 mg, 01.63 mmol), KO*^t^*Bu (160 mg, 1.71 mmol) in THF (10 mL)) and 1,2-dichlorotetramethyldisilane (0.854 mmol, 16 mg). After 5 h same work up procedure as for **5** yielded colorless crystalline **14** (830 mg, 95%) after recrystallization using pentane/acetone. ^1^H-NMR (δ in ppm, CDCl_3_): 0.46 (s, 12H), 0.42 (s, 12H), 0.35 (s, 12H), 0.30 (s, 36H), 0.28 (s, 6H), 0.20 (s, 36H). ^29^Si-NMR (δ in ppm, CDCl_3_): −9.2 (Me_3_*Si*SiMe), −11.7 (Me_3_Si), −28.2 (SiMe_2_), −31.2 (SiMe_2_), −31.6 (SiMe_2_), −80.6 (*Si*Me), −118.3 ((Me_3_Si)_2_*Si*). UV absorption: λ = 265 nm (ε = 5.0 × 10^4^ M^−1^·cm^−1^).

*2,5-Bis(trimethylsilyl)-2,5-bis(dimethyl-2’-exo-norbornylsilyl)decamethylhexasilane* (**15**). A solution of tris(trimethylsilyl)silyl potassium (prepared from: tetrakis(trimethylsilyl)silane (2000 mg, 6.23 mmol) and KO*^t^*Bu (721 mg, 6.42 mmol) in DME (40 mL) at 60 °C) was added dropwise to a DME (10 mL) solution of dimethyl-2-*exo*-norbornylchlorosilane (1294 mg, 6.85 mmol). After 24 h same work up procedure as for **5** and crystallization out of acetone at rt. yielded colorless crystalline 2-(dimethyl-2’-*exo*-norbornylsilyl)-2-(trimethylsilyl)hexamethyltrisilane (**15a**) (1285 mg, 51%). Mp.: 55–60 °C. ^1^H-NMR (δ in ppm): 2.31 (s, 2H) 1.75–1.13 (m, 9H), 0.26 (s, 27H, 3 SiMe_3_), 0.21 (s, 3H, SiMe_2_), 0.16 (s, 3H, SiMe_2_). ^13^C-NMR (δ in ppm): 38.8, 37.7, 37.1, 34.2, 33.5, 30.5, 28.8, 2.9 (3 SiMe_3_), −0.3 (SiMe_2_) −0.5 (SiMe_2_). ^29^Si-NMR (δ in ppm): −5.6 (SiMe_2_), −10.8 (SiMe_3_), −136.3 (Si_q_). Anal. calcd. for C_18_H_44_Si_5_ (400.97): C 53.92, H 11.06. Found: C 53.61, H 10.96.

A solution of 2-dimethyl-2’-*exo-*norbornylsilylhexamethyltrisilyl-2-potassium (prepared from **15a** (1285 mg, 3.20 mmol) and KO*^t^*Bu (377 mg, 3.36 mmol) in THF (40 mL) at rt.) is slowly added to a solution of 1,2-dichlorotetramethylsilane (300 mg, 1.6 mmol) in THF (10 mL). After 24 h same work up procedure as for **5** and crystallization out of acetone at −20 °C yielded colorless crystalline **15** (1010 mg, 82%). Mp: 125–127 °C. ^1^H-NMR (δ in ppm): 2.50 (s, 4H) 1.62–0.90 (m, 18H), 0.57 (s, 12H, SiMe_2_), 0.34 and 0.33 (each s, 18H, SiMe_3_), 0.31 and 0.24 (each s, 6H, SiMe_2_). ^13^C-NMR (δ in ppm): 39.9, 38.9, 37.1, 34.1, 33.8, 32.3, 28.8, 3.8 (SiMe_3_), 1.8 (2 SiMe_3_), 0.4 and 0.6 (SiMe_2_). ^29^Si-NMR (δ in ppm): −5.2 (SiMe_2_), −9.7 (2 SiMe_3_), −29.1 (2 SiMe_2_), −127.4 (Si_q_). Anal. calcd. for C_34_H_82_Si_10_ (771.88): C 52.91, H 10.71. Found: C 52.41, H 10.59. UV absorption: λ = 258 nm (ε = 7.1 × 10^4^ M^−1^·cm^−1^).

*2,5-Bis(trimethoxysilyl)-2,5-bis(trimethylsilyl)decamethylhexasilane* (**16**). Tris(trimethyl-silyl)]-trimethoxy-silylsilane (100 mg, 0.272 mmol) and KO*^t^*Bu (32 mg, 0.285 mmol) were dissolved in THF (2 mL). After NMR spectroscopy confirmed complete formation of the silanide **16a** the solution was added dropwise to an ice cooled solution of 1,2-dichlorotetramethyldisilane (27 mg, 0.142 mmol) in toluene (5 mL). After 2 h the solvent was removed and the residue crystallized from a mixture of diethylether and acetonitrile 2:1. Colorless crystals of **16** (67 mg, 69%) were obtained. Mp: 134–138 °C. ^1^H-NMR (δ in ppm): 3.48 (s, 18H, OMe_3_), 0.71 (s, 12H, SiMe_2_), 0.46 (s, 36H, SiMe_3_). ^13^C-NMR (δ in ppm): 50.3 (OMe), 3.3 (SiMe_3_), 0.4 (SiMe_2_). ^29^Si-NMR (δ in ppm): −9.2 (SiMe_3_), −30.5 (SiMe_2_), −32.7 (Si(OMe)_3_), −135.7 (SiMe_3_). Anal. calcd. for C_22_H_66_O_6_Si_10_ (706.25): C 37.34, H 9.40. Found: C 38.48, H 9.00. UV absorption: λ = 253 nm (ε = 8.4 × 10^4^ M^−1^·cm^−1^) in n-hexane.

For the characterization of silanide **16a** its formation was undertaken in the presence of 18-crown-6: A mixture of 50 mg (0.135 mmol, 1 eq) of tris(trimethyl-silyl)]-trimethoxy-silylsilane, KOtBu (16 mg, 0.142 mmol) and 18-crown-6 (38 mg, 0.098 mmol) was dissolved in C_6_D_6_ (1 mL) and left for 14 h. After NMR spectroscopy confirmed formation of methoxysilylpotassium in a pale beige solution, C_6_D_6_ was removed in vacuum after which the residue was dissolved in diethylether. Very sensitive pale beige crystals of **16a** were obtained on the walls of the vial. Yield: (80 mg, 100%). ^1^H-NMR (d in ppm: 3.74 (s, 9H, OCH_3_), 3.24 (s, 24H, CH_2_O), 0.68 (s, 18H, Si(CH_3_)_3_). ^13^C-NMR (d in ppm): 70.1 (CH_2_O), 50.1 (OMe), 7.7 (SiMe_3_). ^29^Si-NMR (d in ppm): 0.9 (Si(OMe)_3_), −4.3 (Si(*Si*Me_3_)_3_); −220.9 (*Si*(SiMe_3_)_3_).

*2,5-Bis(tert-butyldiphenylsilyl)-2,5-bis(trimethylsilyl)decamethylhexasilane* (**17**). Compound **23** (300 mg, 0.616 mmol) and KO*^t^*Bu (71 mg, 0.635 mmol) were dissolved in THF (3 mL). After NMR spectroscopy confirmed complete formation the solvent was removed, the residue solved in toluene (3 mL) and added dropwise over 5 minutes to a solution of 1,2-dichlorotetramethyldisilane (60 mg, 0.323 mmol) in toluene (2 mL). After 3 h same work up procedure as for **5** yielded after recrystallization from pentane/acetone 2:1 colorless crystalline 25 (210 mg, 72%). Mp: 200–204 °C. ^1^H-NMR (δ in ppm CDCl_3_): 7.66 (m, 8H, Ph), 7.35 (m, 12H, Ph), 1.09 (s, 18H, *^t^*Bu), 0.47 (s, 12H, SiMe_2_), 0.11 (s, 36H, SiMe_3_). ^13^C-NMR (δ in ppm CDCl_3_): 137.38, 137.17, 128.81, 127.16, 29.77 (*^t^*Bu), 20.39 (*^t^*Bu), 4.54 (SiMe_3_), 2.84 (SiMe_2_). ^29^Si-NMR (δ in ppm CDCl_3_): 5.4 (SiPh_2_*^t^*Bu), −8.7 (Me_3_Si), −23.8 (Me_2_Si), −121.9 (Si_q_). Anal. calcd. for C_48_H_86_O_6_Si_10_ (944.07): C 61.07, H 9.18. Found: C 60.94, H 8.93. UV absorption: λ = 240 nm (ε = 3.2 × 10^4^ M^−1^·cm^−1^), λ = 270 nm (ε = 6.8 × 10^4^ M^−1^·cm^−1^) in diethylether.

*2,2,5,5-Tetrakis(trimethylsilyl)-3,4-diphenyloctamethylhexasilane* (**18**). To a solution of 1,2-dimethyltetraphenyldisilane (775 mg, 1.9 mmol) in CH_2_Cl_2_ (10 mL) triflic acid (604 mg, 3.9 mmol) is added slowly. After 12 h the reaction is complete (controlled by ^29^Si-NMR), the solvent is removed and the white precipitate is suspended in toluene (10 mL). A solution of tris(trimethylsilyl)silyl potassium (prepared from tetrakis(trimethylsilyl)silane (1192 mg, 3.7 mmol), KO*^t^*Bu (3.9 mmol), and 18-crown-6 (3.9 mmol) in toluene (10 mL)) is added slowly. After 24 h same work up procedure as for **5** and crystallization out of acetone at −20 °C yielded colorless crystalline **18** (1.43 g, 98%). Mp: 271–273 °C. ^1^H-NMR (δ in ppm): 6.70 (m, 10H), 0.39 (s, 6H, (SiMe)_2_), 0.30 (s, 54H, (SiMe_3_)_6_). ^13^C-NMR (δ in ppm, CDCl_3_): 138.8, 135.7, 128.2, 127.9, 3.1 (SiMe_3_), −0.6 (SiMePh). ^29^Si-NMR (δ in ppm): −9.5 (SiMe_3_), −30.3 (SiMePh), −124.2 (*Si_q_*). Anal. calcd. for C_32_H_70_Si_10_ (735.76): C 52.24, H 9.59. Found: C 49.73, H 9.36. UV absorption: λ = 262 nm (ε = 2.4 × 10^4^ M^−1^·cm^−1^).

*1,5-Bis(pentamethyldisilanyl)-1,1,5,5-tetrakis(trimethylsilyl)hexamethylpentasilane* (**19**). To a solution of pentamethyldisilanylchloride (260 mg, 1.559 mmol) in benzene (4 mL) a solution of 2,6-bis(trimethylsilyl)dodecamethylheptasilyl 2,6-dipotassium (prepared from: 2,2,6,6-tetrakis(trimethylsilyl)dodecamethylheptasilane (390 mg, 0.773 mmol), 18-crown 6 (413 mg, 2.02 equiv) and KO*^t^*Bu (175 mg, 2.02 equiv) in benzene (5 mL)) was added dropwise over a period of 10 min. After 2 h same work up procedure as for **5** and colorless waxy **19** (528 mg, 87%) was obtained. Mp: 85–95 °C. ^1^H-NMR (δ in ppm): 0.54 (s, 12H + 6H), 0.40 (s, 12H), 0.36 (s, 36H), 0.22 (s, 18H). ^13^C-NMR (δ in ppm): 4.0, 1.6, −0.3, −0.4, −1.6. ^29^Si-NMR (δ in ppm): −9.6, −14.4, −29.7, −36.7, −39.0, −122.5. Anal. calcd. for C_28_H_84_Si_13_ (786.083): C 42.78, H 10.77. Found: C 41.23, H 9.99. UV absorption: λ_1_ = 226 nm (ε_1_ = 2.1 × 10^4^ M^−1^·cm^−1^), λ_2_ = 230 nm (ε_2_ = 1.9 × 10^4^ M^−1^·cm^−1^), λ_3_ = 273 nm (ε_2_ = 3.6 × 10^4^ M^−1^·cm^−1^).

*2,2,8,8-Tetrakis(trimethylsilyl)hexadecamethylnonasilane* (**20**). A solution of **19** (210 mg, 0.272 mmol) and Al(Fe)Cl_3_ (20 mg) in cyclohexane (3 mL) was heated to reflux. After 16 h the reaction was complete (controlled by ^29^Si-NMR) and acetone (10 mL) was added. The precipitate was removed by centrifugation, the solvent removed and the residue crystallized by using acetone. Colorless crystalline of **20** was obtained (136 mg, 65%). Mp: 182–186 °C. ^1^H-NMR (δ in ppm): 0.49 (s, 12H), 0.43 (s, 12H), 0.39 (s, 6H), 0.33 (s, 54H). ^13^C-NMR (δ in ppm): 3.4, 0.9, −2.7, −3.3. ^29^Si-NMR (δ in ppm): −9.8, −31.9, −35.3, −36.6, −129.1. Anal. calcd. for C_28_H_84_Si_13_ (786.083): C 42.78, H 10.77. Found: C 41.23, H 9.99. UV Absorption: λ_1_ = 288 nm (ε_1_ = 4.0 × 10^4^ (M^−1^·cm^−1^)), λ_2_ = 271 nm (ε_2_ = 2.7×10^4^ M^−1^·cm^−1^), λ_3_ = 246 nm (ε_3_ = 1.7 × 10^4^ M^−1^·cm^−1^).

*1,4-Bis(triisopropylsilyl)-1,4-bis(trimethylsilyl)octamethylcyclohexasilane* (**21**). A dark red solution of the dianion (prepared from: **9** (215 g, 0.276 mmol), KO*^t^*Bu (68 mg, 0.606 mmol), 18-crown-6 (160 mg, 0.606 mmol) in benzene (10 mL)) diluted with THF (3 mL) was added dropwise to a solution of 1,2-dichlorotetramethyldisilane (48 mg, 0.256 mmol) in THF (5 mL). After 24 h same work up procedure as for **5** yielded colorless crystalline **21** (80 mg, 39%) after recrystallization with Et_2_O. Anal. calcd. for C_32_H_84_Si_10_ (749.86): C 51.25, H 11.29. Found: C 51.01, H 11.03. The crystals were so large that they could be separated manually and so it was possible to characterize both isomers.

*Cis-1,4-bis(triisopropylsilyl)-1,4-bis(trimethylsilyl)cyclohexasilane* (**21a**): Mp: 229–231 °C. ^1^H-NMR (δ in ppm): 1.35 (sept, 6H, Si(C*H*)(CH_3_)_2_), 1.20 (d, *J* = 7.2 Hz, 36H, Si(CH)(C*H*_3_)_2_), 0.48 (s, 12H, SiMe_3_), 0.47 (s, 12H, SiMe_3_), 0.40 (s, 18H, SiMe_3_). ^13^C-NMR (δ in ppm): 20.6 (*^i^*Pr), 14.6 (*^i^*Pr), 5.4 (SiMe_3_), 1.2 (SiMe_3_), 0.9 (SiMe_2_). ^29^Si-NMR (δ in ppm): 22.8 (Si-*^i^*Pr_3_), −8.7 (SiMe_3_), −37.0 (SiMe_2_), −128.9 (Si_q_). UV absorption: λ_1_ = 251 nm (ε_1_ = 6.1 × 10^3^ M^−1^·cm^−1^), shoulder: 276 nm (ε = 8.3 × 10^2^ M^−1^·cm^−1^). 

*Trans-1,4-bis(triisopropylsilyl)-1,4-bis(trimethylsilyl)cyclohexasilane* (**21b**): Mp: 240–242 °C. ^1^H-NMR (δ in ppm): 1.40 (sept, 6H, Si(C*H*)(CH_3_)_2_), 1.21 (d, *J* = 7.2 Hz, 36H, Si(CH)(C*H*_3_)_2_), 0.53 (s, 12H, SiMe_2_), 0.46 (s, 12H, SiMe_2_), 0.40 (s, 18H, SiMe_3_). ^13^C-NMR (δ in ppm): 20.5 (*^i^*Pr), 14.8 (*^i^*Pr), 5.5 (SiMe_3_), 1.6 (SiMe_2_), 0.8 (SiMe_2_). ^29^Si-NMR (δ in ppm): 17.2 (Si-*^i^*Pr_3_), −9.3 (SiMe_3_), −35.3 (SiMe_2_), −127.8 (Si_q_). UV absorption: λ_1_ = 250 nm (ε_1_ = 8.8 × 10^3^ M^−1^·cm^−1^), shoulder: 267 nm (ε = 3.2 × 10^3^ M^−1^·cm^−1^).

*1,1,4,4-Tetrakis(trimethylsilyl)-2,3-diphenyltetramethylcyclopentasilane* (**22**). A benzene (5 mL) solution of **18** (283 mg, 0.38 mmol), KO*^t^*Bu (88 mg), and 18-crown-6 (208 mg) is stirred for 12 h after which the conversion to 2,5-bis(trimethylsilyl)-3,4-diphenyloctamethylhexasilyl 2,5-dipotassium (**18a**) is complete as could be proved by NMR. (**18a**: ^1^H-NMR (δ in ppm): 6.70 (m, 10H), 1.11 (s, 6H, SiMePh), −0.03 (s, 36H, SiMe_3_). ^29^Si-NMR (δ in ppm): −5.0 (SiMe_3_), −22.7 (SiMePh), −180.5 (Si_q_).)

This solution was added dropwise to dichlorodimethylsilane (45 mg, 0.35 mmol) in toluene (2 mL). After 24 h same work up procedure as for **5** and crystallization out of acetone at rt. yielded colorless crystalline **22** (213 mg, 85%). Mp: 194–197 °C. ^1^H-NMR (δ in ppm): 7.00–7.70 (mp, 10H), 0.81 (s, 6H, SiMePh), 0.64 (s, 6H, SiMe_2_), 0.36 (s, 18H, (SiMe_3_), 0.14 (s, 18H, SiMe_3_). ^13^C-NMR (δ in ppm): 138.7, 135.4, 128.3, 127.8, 4.2 (SiMePh), 4.1 (SiMe_3_), 3.5 (SiMe_2_), −1.1 (SiMe_2_). ^29^Si-NMR (δ in ppm): −6.6 (SiMe_3_), −8.1 (SiMe_3_), −23.4 (SiMePh), −20.7 (SiMePh), −127.6 (Si_q_). Anal. calcd. for C_28_H_58_Si_9_ (647.54): C 51.94, H 9.03. Found: C 50.73, H 8.86. UV absorption: shoulder: λ = 256 nm (ε = 1.0 × 10^4^ M^−1^·cm^−1^).

*2-(tert-Butyldiphenylsilyl)-2-(trimethylsilyl)hexamethyltrisilane* (**23**). Tetrakis(trimethyl-silyl)-silane (2.00 g, 6.23 mmol), KO*^t^*Bu (721 mg, 6.41 mmol) and 18-crown-6 (1.69 g, 6.41 mmol) were dissolved in toluene (20 mL). After NMR showed complete formation of hypersilyl potassium, this orange solution was added dropwise to *tert*-butylchlorodiphenylsilane in toluene (10 mL) at −60 °C. The reaction mixture was allowed to warm to room temperature. After 6 h same work up procedure as for **5** and crystallization out of acetone yielded colorless crystalline **23** (3.25 g, 69%). Mp: 342–345 °C. ^1^H-NMR (δ in ppm, CDCl_3_): 7.64 (m, 4H), 7.35 (m, 6H), 1.10 (s, 9H, (*^t^*Bu), 0.12 (s, 27H, (Me_3_Si).^13^C-NMR (δ in ppm, CDCl_3_): 137.2, 137.1, 128.7, 127.2, 29.4 (*Me_3_*-C), 20.2 (Me_3_-*C*), 3.6 (*Me_3_*Si). ^29^Si-NMR (δ in ppm, CDCl_3_): 3.7 (*^t^*BuPh_2_***Si***), −9.4 (Me_3_***Si***), −134.1 ((Me_3_Si)_3_***Si***). Anal. calcd. for C_25_H_46_Si_5_ (487.07): C 61.65, H 9.52. Found: C 60.93, H 9.38. UV absorption: λ = 239 nm (ε = 1.22 × 10^4^ M^−1^·cm^−1^).

*2-(2’-exo-Norbornyldimethylsilyl)-2-(triisopropylsilyl)hexamethyltrisilane* (**24**). A solution of tris(trimethylsilyl)triisopropylsilylsilane (639 mg, 1.58 mmol) and KO*^t^*Bu (186 mg 1.66 mmol) in DME (10 mL) was stirred at 60 °C for 1h. This solution was transferred into a dropping funnel and added slowly to a solution of dimethyl-2-*exo*-norbornylchlorosilane in DME (5 mL). After 18 h, the same work-up procedure as for **5** yielded a colorless oil of **24** (529 mg, 69%). Crystallization with acetone gave colorless plates suitable for X-ray analysis. Mp: 110–112 °C. ^1^H-NMR (δ in ppm): 1.50–2.50 (m, 11H, C_7_*H*_11_Si), 1.28 (sept, 3H, SiC*H*(CH_3_)_2_), 1.18 (d, ^2^*J*_HH_ = 6.9 Hz, 18H, SiCH(C*H*_3_)_2_), 0.34 and 0.33 (s, each 9H, Si(C*H*_3_)_3_) 0.31 and 0.25 (s, each 3H, SiMe_2_). ^13^C-NMR (δ in ppm): 39.1, 38.0, 37.0, 34.0, 33.9, 29.8, 28.9 (Si(CH_3_)_2_*C*_7_H_11_), 20.2 (SiCH(*C*H_3_)_2_), 14.8 (Si*C*H(CH_3_)_2_), 4.35 and 4.31 (Si(*C*H_3_)_3_), 1.2 and 0.9 (Si(*C*H_3_)_2_). ^29^Si-NMR (δ in ppm): 13.5 (*Si^i^*Pr_3_), −4.9 (*Si*Me_2_), −10.1 (*Si*Me_3_), −134.2 (*Si*Si_4_). Anal. calcd. for C_24_H_56_Si_5_ (485.14): C 59.42, H 11.64. Found: C 58.58, H 11.67. UV absorption: shoulder: 222 nm (ε = 1.5 × 10^4^ M^−1^·cm^−1^), λ_1_ = 274 nm (ε_1_ = 2.6 × 10^3^ M^−1^·cm^−1^), shoulder: 283 nm (ε = 2.3 × 10^3^ M^−1^·cm^−1^).

## 4. Conclusions

One of the interesting aspects of the chemistry of poly- and oligosilanes is the associated property of σ-bond electron delocalization. In recent studies we have shown that terminal tris(trimethylsilyl)silyl groups of short oligosilanes are able to force the molecules to engage in an *all-transoid* conformation, which allows a high degree of σ-bond electron delocalization. In case of longer oligosilanes this directing effect is diminished and a second conformation becomes energetically accessible. When we introduced bulky bis(trimethylsilyl)silylene segments inside the chain as conformational amplifiers, these units produced *cisoid* turns causing rupture of conjugation. The current study is concerned with alteration of the steric bulk of the end groups and internal substituents. By exchange of trimethylsilyl groups against methyl or triisopropyl groups the polysilane chains become either more flexible or more rigid. This change in conformataional behavior is nicely reflected in the shape and position of the UV-absorption bands. While flexible molecules exhibit broad bands, indicating a larger conformational space the rigid molecules show sharp bands which can be associated with the absorption of more precisely defined conformers. The deliberate introduction of phenyl substituents either in terminal or internal positions causes a bathochromic shift of the absorption bands, which is much more pronounced for internal substitution. Subsequent work on these compounds will be concerned with variable temperature studies of these compounds to investigate their thermochromic behavior.

## Figures and Tables

**Table 1:** UV spectroscopic data of some selected oligosilanes.

Compound	Oligosilane Chain Length	λ_1_ [nm]	ε_1_ [M^−1^·cm^−1^]	λ_2_ [nm]	ε_2_ [M^−1^·cm^−1^]	λ_3_ [nm]	ε_3_ [M^−1^·cm^−1^]
***n-*Si_6_Me_14_** [6]	6	260	1.4 × 10^4^				
**8** [10]	6	257	6.6 × 10^4^	-	-	-	-
**2**	6	260	2.4 × 10^4^				
**3** [29]	6	264	3.0 × 10^4^				
**9**	6	264	1.4 × 10^4^				
**15**	6	258	7.1 × 10^4^				
**16**	6	253	8.4 × 10^4^				
**18**	6	262	2.4 × 10^4^				
**17**	6	270	6.8 × 10^4^	240	3.2 × 10^4^		
**25** [10]	7	269	6.2 × 10^4^	-	-	-	-
***n-*Si_12_Me_26_** [28]	12	290					
**5a** [13]	12	~289 (sh)	2.3 × 10^4^	259	7.4 × 10^4^		
**5**	12	268	1.6 × 10^4^	218	3.2 × 10^4^		
**11**	12	264	5.1 × 10^4^	232		216	3.1 × 10^4^
**14**	12	265	5.0 × 10^4^				
**6a** [13]	13	~295 (sh)	1.8 × 10^4^	278	5.6 × 10^4^	258	7.3 × 10^4^
**6**	13	~287 (sh)	1.3 × 10^4^	272	1.7 × 10^4^	212	2.7 × 10^4^
**12**	13	~307 (sh)	3.1 × 10^3^	~283 (sh)	2.6 × 10^4^	267	4.6 × 10^4^
**7a** [13]	14	~303 (sh)	1.8 × 10^4^	286	7.8 × 10^4^	259	9.5 × 10^4^
**7**	14	285	2.4 × 10^4^	222	3.6 × 10^4^		
**13**	14	~311 (sh)	1.5 × 10^4^	293	3.8 × 10^4^	268	4.5 × 10^4^
**27** [13]	9	~284 (sh)	1.2 × 10^4^	262	9.2 × 10^4^		
**19**	9	273	3.6 × 10^4^	230	1.9 × 10^4^	226	2.1 × 10^4^
**20**	9	288	4.0 × 10^4^	271	2.7 × 10^4^	246	1.7 × 10^4^

**Table 2:** Conformational properties of oligosilanes described as the torsional angles of the main chain.

Compound	ω_1_	ω_2_	ω_3_	ω_4_	ω_5_	Conformation
**8** [10,37]	159	180	159			TAT
**9**	168	180	168			TAT
**14**	178	158	−83	159	180	ATOTATOTA
**18**	162	180	162			TAT
**15**	160/161	180	160/161			TAT
**17**	160/160	180	160/160			TAT

## Supplementary Materials

The following are available online at http://www.mdpi.com/1420-3049/21/8/1079/s1. Crystallographic information for compounds **9**, **14**, **15**, **16a**, **17**, **18**, **18a**, **21a**, **21b**, **22**, **23**, and **24** in CIF format and crystallographic tables.
