# Supplementary material for: σ-Bond Electron Delocalization in Oligosilanes as Function of Substitution Pattern, Chain Length, and Spatial Orientation
Source: Molecules. 2016 Aug 18;21(8):1079. doi: 10.3390/molecules21081079 (PMC6273834; doi:10.3390/molecules21081079)
Supplement: Supplementary file 1 [file molecules-21-01079-s001.pdf]

Supplementary Materials: σ-Bond Electron Delocalization in Oligosilanes as Function of Substitution Pattern, Chain Length, and Spatial Orientation

Johann Hlina, Filippo Stella, Mohammad Aghazadeh Meshgi, Christoph Marschner and Judith Baumgartner

Table S1. Crystallographic data for compounds **9**, **14**, **15**, and **16a**.

	**9**	**14**	**15**	**16a**
Empirical formula	$Si_{10}C_{34}H_{90}$	$Si_{18}C_{38}H_{114}$	$Si_{10}C_{34}H_{82}$	$Si_4C_{21}H_{51}KO_9$
M_w	779.96	1076.91	771.90	599.08
Temperature [K]	100(2)	100(2)	100(2)	100(2)
Size [mm]	0.44 × 0.34 × 0.18	0.32 × 0.22 × 0.20	0.40 × 0.24 × 0.10	0.22 × 0.15 × 0.12
Crystal system	monoclinic	triclinic	triclinic	orthorhombic
Space group	C2/c	P-1	P-1	Pbca
a [Å]	19.449(4)	9.941(2)	9.603(2)	17.927(6)
b [Å]	9.1445(2)	14.054(3)	15.262(3)	18.514(7)
c [Å]	28.606(6)	15.042(3)	18.530(4)	19.920(7)
α [°]	90	117.16(3)	106.80(3)	90
β [°]	103.34(3)	96.81(3)	102.56(3)	90
γ [°]	90	103.20(3)	106.32(3)	90
V [$Å^3$]	4950.0(2)	1759(2)	2360(2)	6612(4)
Z	4	1	2	8
ρ_{calc} [gcm^{-3}]	1.046	1.017	1.086	1.204
Absorption coefficient [mm^{-1}]	0.287	0.346	0.300	0.346
F(000)	1736	594	852	2592
θ range	1.46 < θ < 26.37	1.57 < θ < 23.50	1.21 < θ < 26.37	1.88 < θ < 25.25
Reflections collected/unique	19231/5066	11205/5173	16509/8754	43966/5979
Completeness to θ [%]	99.9	99.5	96.8	100
Data/restraints/parameters	5066/0/213	5173/0/272	8754/0/417	5979/0/325
Goodness of fit on F^2	1.07	1.04	1.14	1.26
Final R indices [I > 2σ(I)]	R1 = 0.034, *w*R2 = 0.083	R1 = 0.099, *w*R2 = 0.247	R1 = 0.080, *w*R2 = 0.221	R1 = 0.093, *w*R2 = 0.206
R indices (all data)	R1 = 0.042, *w*R2 = 0.087	R1 = 0.168, *w*R2 = 0.294	R1 = 0.111, *w*R2 = 0.258	R1 = 0.108, *w*R2 = 0.214
Largest diff. Peak/hole [$e^-/Å^3$]	0.50/−0.18	1.44/−0.47	1.54/−1.35	1.25/−0.47

Table S2. Crystallographic data for compounds **17**, **18**, **18a**, and **23**.

	**17**	**18**	**18a**	**23**
Empirical formula	$Si_{10}C_{48}H_{86}$	$Si_{10}C_{32}H_{70}$	$Si_8C_{50}H_{100}K_2O_{12}$	$Si_5C_{25}H_{46}$
M_w	944.07	735.78	1196.22	487.07
Temperature [K]	100(2)	100(2)	100(2)	100(2)
Size [mm]	0.26 × 0.18 × 0.14	0.38 × 0.22 × 0.12	0.48 × 0.36 × 0.28	0.28 × 0.24 × 0.12
Crystal system	triclinic	monoclinic	monoclinic	monoclinic
Space group	P-1	P2(1)/n	Cc	P2(1)/c
a [Å]	13.115(3)	12.147(2)	15.024(3)	10.594(2)
b [Å]	15.143(3)	9.463(2)	20.707(4)	18.139(4)
c [Å]	16.005(3)	22.116(4)	22.101(4)	15.919(3)
α [°]	102.673(3)	90	90	90
β [°]	91.018(3)	100.22(3)	92.50(3)	95.11(3)
γ [°]	113.981(3)	90	90	90
V [$Å^3$]	2813(2)	2502(2)	6869(3)	3047(2)
Z	2	2	4	4
ρ_{calc} [gcm^{-3}]	1.115	0.977	1.157	1.062
Absorption coefficient [mm^{-1}]	0.263	0.281	0.327	0.245
F(000)	1028	804	2584	1064
θ range	1.52 < θ < 26.37	1.79 < θ < 26.35	1.68 < θ < 26.37	1.71 < θ < 26.37
Reflections collected/unique	22690/11347	15247/4995	19475/10423	24118/6202
Completeness to θ [%]	98.5	97.8	94.8	99.5
Data/restraints/parameters	11347/0/545	4995/0/200	10423/2/663	6202/0/283
Goodness of fit on F^2	1.07	1.27	1.15	0.98
Final R indices [I > 2σ(I)]	R1 = 0.050, *w*R2 = 0.119	R1 = 0.064, *w*R2 = 0.132	R1 = 0.065, *w*R2 = 0.159	R1 = 0.048, *w*R2 = 0.108
R indices (all data)	R1 = 0.058, *w*R2 = 0.123	R1 = 0.072, *w*R2 = 0.137	R1 = 0.067, *w*R2 = 0.159	R1 = 0.073, *w*R2 = 0.116
Largest diff. Peak/hole [$e^-/Å^3$]	0.74/−0.31	0.62/−0.33	2.44/−0.42	0.42/−0.25

Table S3. Crystallographic data for compounds **21a**, **21b**, **22**, and **24**.

	**21a**	**21b**	**22**	**24**
Empirical formula	$Si_{10}C_{32}H_{84}$	$Si_{10}C_{32}H_{84}$	$Si_9C_{28}H_{58}$	$Si_5C_{24}H_{56}$
M_w	749.89	749.89	647.55	485.14
Temperature [K]	100(2)	100(2)	100(2)	100(2)
Size [mm]	0.34 × 0.26 × 0.12	0.30 × 0.30 × 0.26	0.38 × 0.27 × 0.18	0.42 × 0.25 × 0.15
Crystal system	monoclinic	monoclinic	monoclinic	monoclinic
Space group	C2/c	C2/c	P2(1)/n	P2(1)/c
a [Å]	16.054(2)	36.810(7)	13.371(3)	19.296(4)
b [Å]	10.615(2)	9.195(2)	20.559(4)	9.368(2)
c [Å]	27.564(5)	31.596(6)	14.604(3)	18.715(4)
$\alpha = \gamma = 90°$; β [°]	99.26(3)	117.88(3)	91.24(3)	114.64(3)
V [Å^3]	4636(2)	9453(3)	4014(2)	3075(2)
Z	4	8	4	4
ρ_{calc} [gcm^{-3}]	1.074	1.054	1.072	1.048
Absorption coefficient [mm^{-1}]	0.304	0.298	0.314	0.242
F(000)	1664	3328	1408	1080
θ range	$1.50 < \theta < 25.00$	$1.25 < \theta < 26.37$	$1.71 < \theta < 26.38$	$2.18 < \theta < 26.35$
Reflections collected/unique	13825/4066	36797/9665	28438/8149	23776/6262
Completeness to θ [%]	99.3	99.8	99.1	99.8
Data/restraints/parameters	4066/0/203	9665/0/405	8149/0/350	6262/0/282
Goodness of fit on F^2	1.30	1.09	1.03	1.04
Final R indices [$I > 2\sigma(I)$]	R1 = 0.095, *w*R2 = 0.184	R1 = 0.035, *w*R2 = 0.083	R1 = 0.046, *w*R2 = 0.123	R1 = 0.070, *w*R2 = 0.173
R indices (all data)	R1 = 0.112, *w*R2 = 0.190	R1 = 0.040, *w*R2 = 0.085	R1 = 0.056, *w*R2 = 0.127	R1 = 0.073, *w*R2 = 0.176
Largest diff. Peak/hole [e$^-$/Å^3]	0.82/−0.49	0.47/−0.21	0.57/−0.35	2.23/−1.40
